# The p40 fusion domain as a scaffold for producing functional inclusion bodies

**DOI:** 10.1007/s00253-026-13810-4

**Published:** 2026-04-18

**Authors:** Santhosh Vijayakumar, Riya Khetan, Anwar Sunna

**Affiliations:** 1 School of Natural Sciences, Macquarie University, North Ryde, Sydney, NSW 2109 Australia; 2 Australian Research Council Industrial Transformation Training Centre for Facilitated Advancement of Australia’s Bioactives (FAAB), Sydney, NSW 2109 Australia; 3 Centre for Pharmaceutical Innovation (CPI), Clinical & Health Science, University of South Australia, Adelaide, SA 5000 Australia

**Keywords:** Biocatalysis, Enzyme immobilisation, Recyclable biocatalysts, Protein aggregation, Fusion tags, Antimicrobial peptide

## Abstract

**Abstract:**

Inclusion bodies (IBs) in *Escherichia coli* were once regarded as undesirable aggregates of misfolded proteins, but are now increasingly recognised as functional biomaterials. Here, we show that the N-terminal p40 domain of a lytic polysaccharide monooxygenase from *Caldibacillus cellulovorans* acts as a scaffold for producing functional inclusion bodies. This property was demonstrated across diverse proteins, including the fluorescent protein mCherry, three industrially relevant enzymes, and the antimicrobial peptide ZXR-2. The p40 domain consistently promoted IB formation under a wide range of induction conditions, and the resulting protein nanoparticles retained enzymatic activity, could be reused over multiple reaction cycles, and, when fused to ZXR-2, exhibited both antimicrobial and cytotoxic activities. Biophysical analyses revealed that IB size and morphology were influenced by cultivation parameters, highlighting the tunability of p40-mediated assemblies. Comparative analyses underscored the structural robustness of p40-driven IBs and their ability to support diverse protein contexts. While not intended for direct therapeutic use, these results emphasise the potential of p40 IBs as a platform technology for in vitro studies, biocatalysis, and other biotechnological applications. These findings establish the p40 fusion domain as a reliable scaffold for functional inclusion body production in *E. coli*, providing a foundation for future applications of IBs as versatile biotechnological tools.

**Key points:**

• *p40 serves as a versatile scaffold for active protein aggregates in E. coli.*

• *Cultivation parameters modulate aggregates accumulation and physical properties.*

• *p40 fusions form recyclable nanobiocatalysts and stabilise antimicrobial peptides.*

**Supplementary Information:**

The online version contains supplementary material available at 10.1007/s00253-026-13810-4.

## Introduction

Inclusion bodies (IBs) were once regarded as inactive aggregates arising during recombinant protein overexpression in *Escherichia coli*, but they are now recognised as functional protein particles with growing relevance in biotechnology. Their high protein content, mechanical robustness, and straightforward recovery make IBs attractive as carrier-free, reusable biomaterials for biocatalysis and related applications (Carrió and Villaverde 2001; Villaverde and Mar Carrió 2003; Gatti‐Lafranconi et al. 2011) .

IBs are intracellular, insoluble assemblies enriched in the overexpressed polypeptide, which often constitutes 50–90% of the IB’s total protein content, and they typically measure 100–800 nm in diameter depending on the protein and process conditions (Morell et al. 2008; Jürgen et al. 2010; Upadhyay et al. 2012; Slouka et al. 2018). Their formation is protein-specific and reflects the kinetics of folding intermediates and host protein homeostasis; IBs can also display dynamic behaviour and, in many cases, retain native-like structure and measurable activity (Carrió and Villaverde 2002; Idicula-Thomas and Balaji 2007). Beyond classical dense, amyloid-like IBs that resist solubilisation, non-classical IBs (ncIBs) are more labile and have been shown to yield folded, active protein under mild extraction conditions. For example, Jevševar and co-workers demonstrated that human granulocyte colony-stimulating factor (hG-CSF) produced in *E. coli* at 25 °C accumulated in ncIBs that were partially soluble, allowing direct purification of functional protein (Jevševar et al. 2008). Although in vitro solubilisation and refolding remain possible routes to recover proteins from IBs, such workflows are generally labour-intensive and empirical, with no universal solution (Vallejo and Rinas 2004; Singh and Panda 2005).

A major advance has been the development of catalytically active IBs (CatIBs), which act as in vivo-assembled immobilisates. CatIBs have been generated for numerous enzymes and shown to function as stable, recyclable biocatalysts (Krauss et al. 2017; Jäger et al. 2019). The most established strategy relies on genetic fusion to aggregation-inducing peptides or domains, including amphipathic helices, hydrophobic self-assembling motifs, viral capsid proteins, coiled-coil domains, and carbohydrate-binding modules (Rehm et al. 2016; Köszagová et al. 2022). In downstream processing, CatIBs can be recovered by standard mechanical disruption (e.g., high-pressure homogenisation or controlled sonication) and are typically obtained with minimal nucleic acid contamination (Peternel and Komel 2010; Neerathilingam et al. 2014). Their carrier-free nature and reusability make CatIBs economically attractive for industrial biocatalysis, bioremediation, and emerging biomedical applications (Krauss et al. 2017; Ölçücü et al. 2022).

Recent methodological innovations further highlight the momentum of CatIB research. Helleckes et al. (2024) introduced a high-throughput toolbox combining laboratory automation and Bayesian optimisation to streamline the identification of effective fusion partners, thereby accelerating the design of functional IBs. Yet, most studies have focused on enzymes or reporter proteins, whereas small bioactive peptides have received comparatively little attention. Demonstrating that a single fusion domain can drive IB formation for structurally diverse biomolecules would therefore strengthen the concept of IBs as a versatile platform technology.

The p40 domain was first described as the N-terminal region of a multidomain GH5 β−1,4-mannanase (ManA) from *Caldibacillus* *cellulovorans* (Sunna et al. 2000). Initially annotated as a carbohydrate-binding module (CBM33), this domain has since been reclassified as a copper-dependent lytic polysaccharide monooxygenase (LPMO) in the auxiliary activity family AA10 within the CAZy database (Levasseur et al. 2013; Forsberg et al. 2025). In this study, p40 refers to the truncated form lacking the native signal peptide. Our previous observations indicated that recombinant p40 expressed in *E. coli* forms insoluble aggregates. Here, we exploit its intrinsic propensity for self-assembly as a fusion partner to generate functional IBs. We demonstrate that p40 drives IB formation across a fluorescent reporter, two distinct enzymes, and an antimicrobial peptide, thereby establishing proof of principle for p40 as a broadly applicable and minimally optimised fusion domain for producing functional IBs.

## Materials and methods

### Chemicals and reagents

All chemicals and reagents used in this study were purchased from Sigma-Aldrich (Castle Hill, NSW, Australia) unless otherwise specified. Restriction enzymes, T4 DNA ligase, and PCR kits were obtained from New England Biolabs (NEB, Notting Hill, VIC, Australia).

### Construction of expression plasmids

All molecular biology procedures were performed as described by Green and Sambrook (2012). Plasmid pETDuet-1 (Merck) p533, kindly provided by Dr Moreland Gibbs, carried the p40 domain sequence of the multidomain β−1,4-mannanase (ManA) from *Caldibacillus cellulovorans* (Sunna et al. 2000), followed by a short (GGGGS)_3_ linker (L) sequence. The synthesised DNA inserts with their respective restriction sites, and the final recombinant constructs are shown diagrammatically in Table S1. NEB 5-alpha Competent *E. coli* cells were used as the host for plasmid propagation and cloning, while *E. coli* (DE3) or *E. coli* Tuner (DE3) cells (Novagen, EMD Millipore, Billerica, MA, USA) were used for recombinant protein expression. *E. coli* strains were grown in Luria–Bertani (LB) medium supplemented with 100 μg/mL carbenicillin. All inserts were synthesised as codon-optimised gBlock Gene Fragments (Integrated DNA Technologies, Singapore) with appropriate restriction sites and ligated to linearised pETDuet-1_p533 plasmid using the Quick-Stick Ligase protocol (Bioline, Redfern, NSW, Australia). *Dictyoglomus thermophilum* β-xylanase B (xynB) (Morris et al. 1998), *Bacillus subtilis* β-xylanase (BactXyn) (Yoon 2009), *Bacillus licheniformis* α-amylase (BactAmy) (Rasiah and Rehm 2009), and mCherry (UniProt: D1MPT3) inserts were digested with *Nco*l and *Hind*III and ligated into pETDuet-1_p533 previously digested with the same enzymes.

The antimicrobial and anticancer peptide ZXR-2 (Zhou et al. 2016; Chen et al. 2017) was synthesised as a single sequence fused by the linker and p40 sequence with the restriction sites *Pci*I and *EcoR*I, and ligated into pETDuet-1_p533 previously digested with *Nco*I and *EcoR*I.

### Production of p40 IBs

For bare_p40_, XynB_p40_, BactXyn_p40_, BactAmy_p40_, and ZXR-2_p40_ constructs, protein expression was carried out in LB medium supplemented with (100 μg/mL) carbenicillin. Five milliliters of an overnight culture was used to inoculate 500 mL LB medium. Cultures were incubated at 37 °C with shaking (250 rpm) until OD_600_ reached 0.5–0.7. Protein expression was induced with 0.1 mM isopropyl-β-D-thiogalactopyranoside (IPTG), and cells were harvested after 4 h by centrifugation (12,000 × *g* 20 min, 4 °C). Pellets were stored at − 30 °C until further used.

For fluorescent mCherry_p40_, production was performed in Greiner 12-well cell culture plates. Each well was seeded with 3960 μL of mid-log phase culture, and IPTG was added to final concentrations of 0, 0.05, 0.4, and 1 mM. Induction was carried out at 37 °C for 6 h with orbital shaking at 200 rpm, and cells were collected at 1, 3, and 6 h post-induction. Parallel experiments were performed at 26 and 18 °C.

### Purification of p40 IBs

For bare_p40_, XynB_p40_, BactXyn_p40_, BactAmy_p40_, and ZXR-2_p40_ constructs, cell pellets were resuspended in 50 mL lysis buffer (50 mM Tris–HCl, 10 mM NaCl, 1 mM EDTA, pH 7.4) supplemented with either 1 mg/mL lysozyme or 1 U/mL Benzonase® nuclease (Novagen, USA). Cells were disrupted using a CF4 cell disruptor (Constant System Ltd, UK) at 20 kPsi. The lysate was treated with B-PER™ Reagent (ThermoFisher Scientific; 4 mL per g of cell in phosphate buffer) and incubated at room temperature (RT) for 15 min. The insoluble fraction was recovered by centrifugation (12,000 × *g*, 30 min, 4 °C), washed once with a 1:10 dilution of B-PER in sterile deionised water, pelleted again, washed one more, and finally resuspended in 50 mL sterile deionised water. Purified IBs were stored at − 30 °C until further use.

For mCherry_p40_ IBs, cells from 4 mL cultures were harvested by centrifugation (8000 × *g*, 5 min, 4 °C), washed with phosphate-buffered saline (PBS) buffer (pH 7.4), and resuspended in 400 µL of lysis buffer (50 mM Tris–HCl, 100 mM NaCl, 1 mM EDTA, 1 mM Pefabloc, 1% glycerol, pH 8). Cells were disrupted by sonication using a Branson 450 digital sonifier (Marshall Scientific, USA) for 8 min with a 5 s on/10 s off cycle at 40% amplitude. Lysates were centrifuged (12,000 × *g*, 15 min, 4 °C) to separate soluble and insoluble fractions. The pellet containing mCherry_p40_ IBs was washed once with PBS buffer and treated with B-PER® (4 mL per g pellet) supplemented with 5 U benzonase. Samples were incubated at RT for 15 min with gentle agitation and centrifuged again. The pellet was washed twice with diluted B-PER (1:10 in MilliQ water) and once with 0.5% Triton X-100. Purified mCherry_p40_ IBs were stored at − 30 °C until further use.

#### SDS-PAGE

Proteins were analysed by SDS-PAGE using Mini-PROTEAN TGX system (Bio-Rad Laboratories, USA). Samples were mixed with an equal volume of 2 × Laemmli loading buffer and denatured at 95 °C for 5 min. After brief centrifugation, samples were loaded onto pre-cast 4–20% gels and electrophoresed at 100 V for 60 min in running buffer (25 mM Tris, 192 mM glycine, 1% SDS, pH 8.3). Protein bands were visualised by staining with Coomassie Brilliant Blue G-250 safe stain and destaining with MilliQ water. Gel images were acquired using a G:BOX F3 gel documentation system (Syngene, UK). A Precision Plus Protein Dual Colour standard (Bio-Rad) was included in all gels. Densitometric quantification of protein bands was performed using ImageJ software (National Institutes of Health, USA). Fusion protein concentrations were determined by comparing band intensities to a standard curve prepared from carbonic anhydrase (CA) standards (0.01 to 0.3 mg/mL). CA standards and p40 fusion proteins were electrophoresed and stained under identical conditions.

### Protein content estimation of p40 IBs

The molar extinction coefficient and molecular weight of each p40 fusion protein were calculated from the deduced amino acid sequence using the ExPASy ProtParam tool. The total protein content of each IB was determined according to the protocol described by Ölçücü et al. (2023). Briefly, a total of 3 mg of purified IBs was resuspended in 300 μL solubilisation buffer (50 mM Tris–HCl, pH 8.5, 5 mM DTT). Suspensions were pipetted and vortexed vigorously until no visible clumps remained. Aliquots of 100 µL (corresponding to 1 mg IBs) were transferred into three tubes to enable triplicate measurement. Samples were further solubilised by addition of solubilisation buffer containing 3 M guanidinium hydrochloride (GdmCl) to yield a final concentration of 1 mg/mL IBs per tube. After incubation at RT for 1 h with gentle agitation, the samples were centrifuged (12,000 × *g*, 20 min, 4 °C) to remove residual insoluble debris. The absorbance at 280 nm (A_280_) was measured using NanoDrop One/Oneᶜ (Thermo Fisher Scientific) after blanking against solubilisation buffer containing 3 M GdmCl, with baseline correction at 350 nm. Protein concentration was determined applying the Beer-Lambert Law:

$$c\mathrm{Protein} [M]=\frac{\mathrm{A}280\mathrm{nm}}{\varepsilon \mathrm{Protein},A280\mathrm{nm}\times l}$$

where cProtein is the molar concentration (M) of protein, A280 nm is the absorbance at 280 nm; εProtein is the theoretical molar extinction coefficient (calculated with ProtParam), and l- is the pathlength (mm).

The proportion of fusion protein in each IB preparation was calculated as follows:

$$\text{Protein content IBs }\left[\mathrm{\%}\right]=\frac{\text{Protein concentration IBs }[\mathrm{mg}/\mathrm{ml}] }{\text{IB suspension concentration }[\mathrm{mg}/\mathrm{ml}] }\times 100\mathrm{\%}$$

where the IB suspension concentration (mg/mL) corresponds to the wet mass of purified IBs resuspended per volume of solubilisation buffer used in the quantification assay. All the experiments were performed in triplicate, and results are reported as mean values. Solubilisation with GdmCl was performed only on separate aliquots for determination of fusion protein content. All enzymatic and antimicrobial assays were conducted using purified intact IB suspensions.

### Tunable production of p40 IBs

mCherry_p40_ was used as a model to study the tunability of IB production, cellular localisation, and final IB size. The intrinsic fluorescence of mCherry enabled monitoring of expression kinetics, protein yield, and morphological features under different induction conditions.

### Expression kineticsof mCherry_p40_ IBs

Expression kinetics were evaluated by measuring mCherry_p40_ fluorescence under varying conditions, inducer concentration (0–1 mM IPTG), induction time (0–6 h), and temperature (18, 26, and 37 °C). An overnight 5-mL seed culture was grown on LB broth supplemented with 100 µg/mL carbenicillin at 37 °C. Fifty milliliters of LB broth was inoculated with 2% (v/v) of this seed culture and grown at 37 °C until the OD_600_ reached 0.6–0.8 (mid-log phase). Expression kinetics were assessed in a µClear® 96-well flat-bottom microtiter plate (Greiner Bio-One, Austria). Aliquots of 180 µL mid-log phase culture were transferred into individual wells and induced with 20 µL IPTG to achieve final concentrations between 0.05 and 1 mM. Uninduced cultures served controls. Cultivation and fluorescence monitoring were performed in a FLUOstar Omega multi-mode microplate reader (BMG Labtech, Germany) at 18, 26, or 37 °C with double-orbital shaking at 200 rpm. Fluorescence was measured at 5-min intervals using excitation/emission wavelengths of 595/630 nm. All experiments were performed in triplicates, and results are reported as mean ± standard deviation (SD).

### Cellular localisation of mCherry_p40_ IBs

Cells from the expression experiments were collected by centrifugation (4000 × *g*, 3 min, 4 °C). Pellets from 1 mL of culture were washed and resuspended in 200 μL PBS buffer to a final OD_600_ of approximately 1. A 2 μL aliquot of the suspension was pipetted onto an agarose pad on a microscope slide and covered with a glass coverslip. Imaging was performed using an FV3000RS confocal microscope (Olympus, Japan). mCherry_p40_ fluorescence was recorded using excitation/emission filters of 595/630 nm, and images were captured at 100 × magnification. Acquired images were analysed using ImageJ software (National Institutes of Health, USA). Fluorescent IB foci were identified within intact cells, and their apparent size and spatial distribution were assessed qualitatively across multiple fields of view.

### Biophysical characterisation of p40 IBs

#### Electron microscopy

Scanning electron microscopy (SEM) was used to examine the morphology and size of all partially purified p40 IBs, except mCherry_p40_. Samples were washed thoroughly with ultrapure water and passed through a 0.22-µm polycarbonate isopore membrane filter (Merck, USA). The filter was washed three times with ultrapure water, air-dried for 30 min, and gold-coated prior to imaging. SEM images were acquired using a Phenom™ XL G2 system (Phenom Scientific, USA).

For mCherry_p40_ IBs, field emission scanning electron microscopy (FESEM) was performed. Purified IBs were diluted 20-fold using ultrapure water and gentle vortexed. One milliliter of diluted samples was filtered through a 0.22-μm polycarbonate isopore membrane mounted on a 13-mm Swinnex filter holder (Merk, USA). The membrane was rinsed with 500 μL ultrapure water, and excess liquid was removed by passing air through the filter with an empty syringe. After drying under laminar flow, the membrane was mounted on a stub with carbon adhesive tape and sputter-coated with gold using Emitech K550 (Emitech, UK) under argon. Imaging was carried out on a JSM-7100F FESEM (JEOL, Japan) at an accelerating voltage of 5 kV.

The size of p40 IBs was determined by measuring approximately 50 structures per sample using ImageJ software.

#### Dynamic light scattering

Dynamic light scattering (DLS) was performed on a Zetasizer Nano ZSP instrument (Malvern, UK) using a 633-nm laser. Particle size and polydispersity index (PDI) were determined using cumulants analysis. Measurements were carried out at 25 °C in disposable plastic microcuvettes (ZEN0040, Malvern). Samples were prepared by resuspending IB pellets in ultrapure water, 50 mM PBS (pH 6.0), 5 mM HCl (pH 4.1), or 10 mM Tris–HCl (pH 7.4). All buffers were passed through 0.22-μm sterile filters (Millipore, USA) prior to resuspend IBs. Data was processed using Zetasizer Nano software.

### Enzyme assays and recycling

Enzyme activity and recycling experiments were performed at the reported optimum temperature for each enzyme, defined as the temperature corresponding to the highest catalytic activity described in the literature (*D. thermophilum* xylanase: Morris et al. 1998; *B. subtilis* AMX-4 xylanase: Yoon 2009; *B. licheniformis* α-amylase: Rasiah and Rehm 2009). Assays were also conducted at 10 °C below the optimum to evaluate operational stability under slightly suboptimal conditions. Xylanase and amylase activities were measured using the dinitrosalicylic acid (DNS) colorimetric method (Bailey et al. 1992), with reducing sugars released quantified at 540 nm. For xylanase activity, reactions contained 0.5% (w/v) oat spelt xylan (OSX) as substrate in 50 mM sodium phosphate buffer (pH 6.5). For amylase activity, 0.5% (w/v) potato starch was used in 50 mM 4-(2-hydroxyethyl)−1-piperazineethanesulfonic acid (HEPES) buffer (pH 7.8). Standard reaction mixture was prepared to a final volume of 100 μL with substrate and enzyme, and activity was confirmed to be linear with respect to time and enzyme concentration. For the recycling experiments, partially purified insoluble enzyme_p40_ were incubated with substrate under standard assay conditions. After a 10-min incubation, samples were centrifuged briefly to pellet the enzyme_p40_ IBs. The supernatant was transferred to a fresh tube, and the reaction was stopped by addition of 100 μL DNS reagent. Fresh substrate was then added to the pellet, and the assay repeated under the same conditions. Recycling was performed for a total of ten cycles at the selected temperatures.

### Antimicrobial and cytotoxic activities of ZXR-2_p40_ IBs

#### Bacterial killing kinetics

*Lactobacillus fermentum* (IFM 1514) and *L. casei* (IFM 1504) were obtained from IFM Quality Services (Sydney, Australia) and used to assess the antimicrobial activity of ZXR-2_p40_. Briefly, 50 µL of each strain was spread onto Lactobacilli MRS agar (Edwards Group, Sydney) and incubated at 37 °C. A single colony was used to inoculate Lactobacilli MRS broth (Edwards Group), which was incubated at 37 °C without shaking until mid-log phase (OD_570_ between 0.6 and 0.8) was reached. Aliquots of 180 µL of the mid-log phase culture were transferred to wells of a UV-transparent 96-well microplate and supplemented with ZXR-2_p40_ at final concentrations of 1–32 µM in a total volume of 200 µL. Control included wells containing equivalent concentrations of bare_p40_ or untreated cultures. Chemically synthesised ZXR-2 peptide (without p40, > 95% purity) was supplied by Dr Daryl Ariawan (Macquarie University Dementia Research Centre) and used as positive control. Microplates were incubated at 37 °C, and OD_570_ readings were recorded every 6 min over 10 h using a PHERAstar FSX microplate reader (BMG Labtech). All assays were performed in triplicate, and data were analysed mean ± SD. Optical density traces were corrected by subtracting matched IB-only blanks at the corresponding IB concentration to correct for light scattering by insoluble material; conclusions were cross-validated by CFU time-kill assays. Additional assays were performed with *Serratia marcescens* (ATCC 13880, Gram-negative) and *Staphylococcus epidermis* (ATCC 12228, Gram-positive) exposed to 2, 4, and 16 µM of ZXR-2_p40_ under the same conditions.

A time-kill assay was performed to compare the activity of synthesised ZXR-2 peptide and ZXR-2_p40_ against *L. fermentum* and *L. casei*. Mid-log phase cultures were incubated with ZXR-2 (16 µM) and ZXR-2_p40_ (4 µM) in a 1.5-ml microcentrifuge tube at 37 °C without shaking. Aliquots (50 µL) were collected at 0, 5, 15, 30, 60, 120, and 240 min, diluted in 200 µL MRS medium, and spread plated (50 µL) onto Lactobacilli MRS agar. Untreated cultures served as controls. Colony-forming units (CFU/mL) were enumerated after 24-h incubation at 37 °C, and log CFU/mL was plotted against time. All the assays were conducted in triplicates.

To visualise bacterial morphology after treatment, *L. casei* cells in mid-log phase were treated with 4 µM ZXR-2_p40_ and collected at 0, 2, and 4 h. Samples were filtered through a 0.22-µm polycarbonate isopore membrane (Merck), gold sputter-coated, and imaged by SEM using a Phenom™ XL G2 (Phenom Scientific, USA).

#### Cytotoxicity assays

Human embryonic kidney cells HEK-293 T (ATCC® CRL-3216) were used to assess the cytotoxicity of ZXR-2_p40_ IBs. Cells were maintained in Dulbecco’s modified Eagle’s medium (DMEM) supplemented with 10% (v/v) foetal bovine serum (FBS) and 1% (v/v) penicillin–streptomycin at 37 °C in a humidified atmosphere of 95% air and 5% CO_2_. Cells were grown to ~ 90% confluence before use. Cytotoxicity was measured using the 3-(4,5-dimethylthiazol-2-yl)−2,5-diphenyltetrazolium bromide (MTT) assay. Briefly, 5000 cells were seeded per well in 96-well plates and incubated for 24 h at 37 °C to allow monolayer formation. After removal of the culture medium, cells were washed twice with PBS (pH 7.4) and treated with filtered ZXR-2_p40_ samples diluted in culture medium to final concentrations of 5, 10, 15, 20, or 30 µM. Cells were incubated for a further 24 h at 37 °C. Following incubation, media were removed, and cells were treated with 10 µL of MTT solution (5 mg/mL in PBS) for 4 h at 37 °C. Formazan crystals were dissolved in 200 µL dimethyl sulfoxide (DMSO), and absorbance was measured at 570 nm using a PHERAstar FSX microplate reader. All experiments were performed in triplicate, and results are reported as mean ± SD.

## Results

### Expression and purification of bare_p40_ IBs

*E. coli* BL21(DE3) cells harbouring expression plasmid p533 (encoding the p40 domain followed by a short linker sequence) were used to express bare p40 (bare_p40_). Samples collected before and after IPTG induction showed expression of an insoluble protein recovered as IBs following cell disruption and detergent-based purification (Fig. S3). Using the B-PER® extraction protocol, approximately 350 mg of bare_p40_ protein (30–40 mL at 9.2 mg/mL) was partially purified for subsequent studies, with no further optimisation required. SDS–PAGE analysis revealed a major band at ~ 24.4 kDa (Fig. S4), consistent with the calculated molecular weight of 24.6 kDa based on the amino acid sequence.

### Characterisation of bare_p40_IBs

**Fig. 1:**
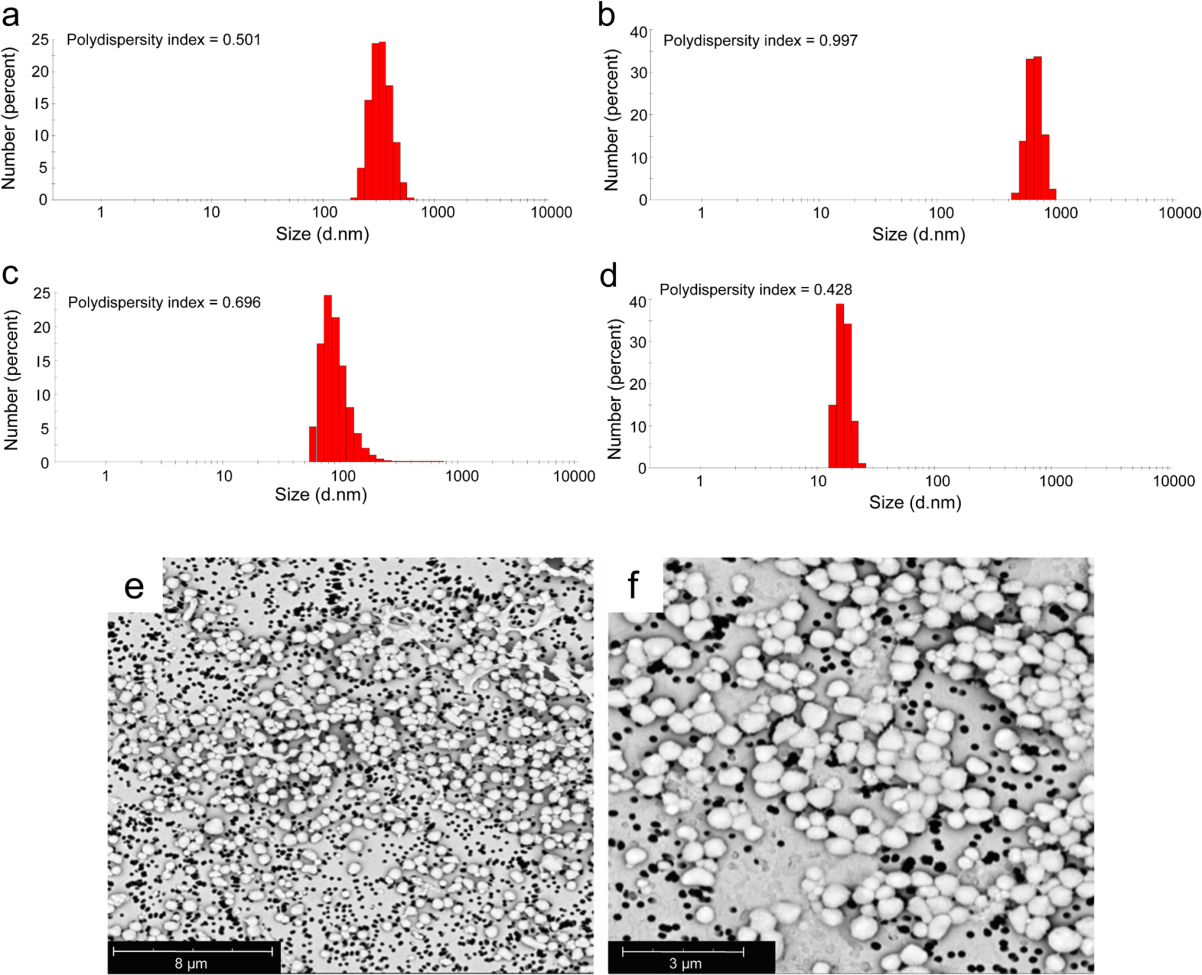
Dynamic light scattering (DLS) data of barep40 resuspended in four different solutions. **a** Ultrapure water. **b** 50 mM PBS, pH 6.0. **c** 5 mM HCl, pH 4.1. **d** 10 mM Tris–HCl, pH 7.4. Scanning electron microscopy (SEM) images of barep40 at magnifications of **e** 10000 × and **f** 20,000 ×

The size distribution of bare_p40_ IBs was analysed by DLS in four different resuspension solutions (Fig. 1). DLS measurements were performed using freshly prepared IB suspensions, and the resulting distributions should therefore be interpreted as relative particle relative hydrodynamic size distributions. Particle sizes ranged from ~ 50 to 1000 nm in most solutions, but in 10 mM Tris–HCl (pH 7.4), smaller particles (10–40 nm) were predominant (Fig. 1d). The polydispersity index (PDI) exceeded 0.5 in most conditions, indicating heterogeneous size distributions. The highest PDI (0.997) was observed in PBS (pH 6.0; Fig. 1b), while the lowest PDI (0.428) was recorded in Tris–HCl (pH 7.4; Fig. 1d), which also produced the smallest particles. The corresponding DLS intensity distribution plot is provided in Fig. S5.

Morphological analysis by SEM (Fig. 1e and f) confirmed that bare_p40_ IBs were present as both individual and aggregated particles in ultrapure water. Most structures were circular, with diameters ranging from 200 to 800 nm, which is broadly consistent with the size range estimated from the DLS measurements.

### The effect of production parameters on p40 IBs

Fluorescent protein fusions are widely used as model systems because fluorescence provides a direct proxy for expression kinetics and aggregation behaviour (Raghunathan et al. 2014; Ölçücü et al. 2022). Accordingly, the mCherry_p40_ fusion construct was employed here to evaluate the tunability of p40-mediated IB formation under systematically varied induction conditions.

### Expression kinetics of mCherry_p40_ IBs

**Fig. 2:**
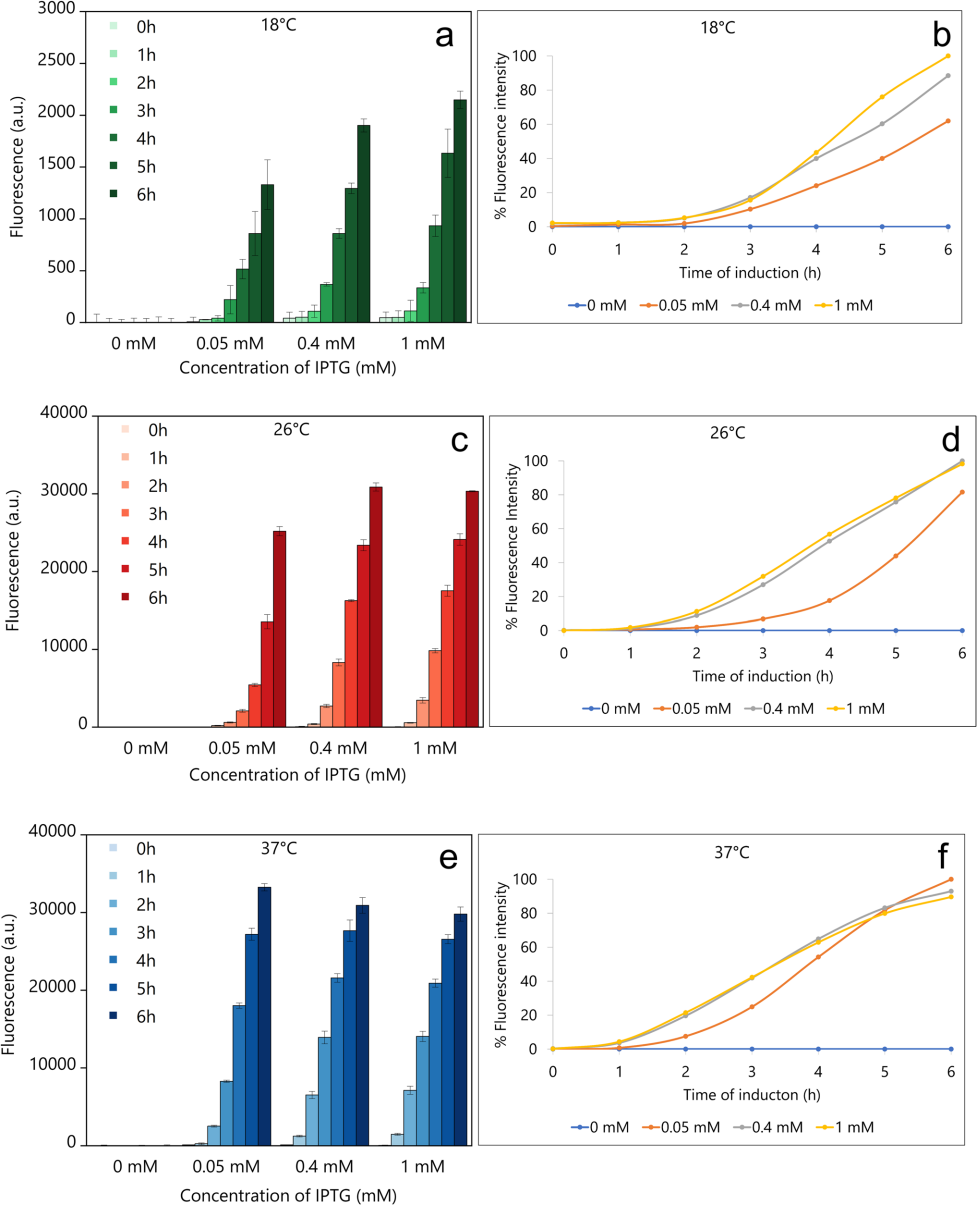
Expression kinetics of mCherry_p40_ IBs in *E. coli* Tuner (DE3) under varying induction conditions. Fluorescence was monitored over 6 h at IPTG concentrations of 0.05, 0.4, and 1 mM for each temperature. Panels **a**, **c**, and **e** display raw fluorescence intensity (FI) at 18, 26, and 37 °C, respectively; panels **b**, **d**, and **f** show corresponding normalised percentage FI at the same temperatures, calculated by subtracting baseline FI from non-induced controls

mCherry_p40_ expression was studied in *E. coli* Tuner (DE3), a BL21(DE3) derivative lacking the lactose permease gene (lacZY), which ensures homogeneous IPTG uptake and concentration-dependent expression. Expression kinetics were assayed at two sub-optimal (18 and 26 °C) and at the optimal growth temperature for *E. coli* (37 °C), using IPTG concentrations of 0.05, 0.4, and 1 mM over a 6-h induction period (Fig. 2).

At 18 °C, mCherry fluorescence intensity (FI) remained very low for the first 2 h at all IPTG concentrations (Fig. 2a). After 3 h, FI increased exponentially, reaching a maximum after 6 h induction. Cells induced with 1 mM IPTG showed the highest FI, while those induced with 0.4 and 0.05 mM IPTG displayed 88% and 62%, respectively, of the 1 mM maximum (Fig. 2b). The final cell density (OD_600_) at 6 h increased only modestly (0.35–0.4-fold) compared with the initial OD_600_ (data not shown). At 26 °C, cells also showed low FI during the first 2 h post-induction (Fig. 2c). Thereafter, FI increased exponentially at all IPTG concentrations, reaching a maximum at 6 h. Cells induced with 0.4 and 1 mM IPTG displayed nearly identical FI profiles, both reaching the same maximum after 6 h. In contrast, cells induced with 0.05 mM IPTG reached only 82% of this maximum (Fig. 2d). The final OD_600_ increased by 1.8-fold (0.05 mM) and 2.8- to 3.0-fold (0.4 and 1 mM; data not shown). At 37 °C, the optimal growth temperature for *E. coli*, induction proceeded faster, with FI increasing exponentially from 2 h post-induction (Fig. 2e). At all IPTG concentrations, the hourly FI profiles were very similar across the 6-h period. Cells induced with 0.05 mM IPTG reached the highest FI at 6 h, while those induced with 0.4 and 1 mM IPTG reached 93% and 90%, respectively, of this value (Fig. 2f). The final OD_600_ increased by 2.6-fold (0.05 mM) and by 2.2- and 2.0-fold at 0.4 and 1 mM IPTG, respectively (data not shown), indicating a slightly negative effect of higher IPTG concentrations on cell growth.

### Yield of mCherry_p40_ IBs at different growth and expression conditions

**Fig. 3:**
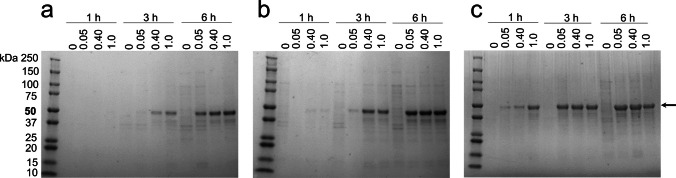
Yield and purity of mCherry_p40_ IBs analysed by SDS-PAGE. IB samples were produced at **a** 18 °C, **b** 26 °C, and **c** 37 °C at IPTG concentrations of 0.05, 0.4, and 1 mM and harvested after 1, 3, and 6 h post-induction. mCherry_p40_ protein bands (~ 50.6 kDa) are indicated by an arrow. Gels were stained with Coomassie blue

The production of mCherry_p40_ IBs was investigated under varying post-induction temperatures (18, 26, and 37 °C), IPTG concentrations (0–1 mM), and induction times (1–6 h). SDS–PAGE analysis revealed that at 18 °C, no visible mCherry_p40_ band (~ 50.6 kDa) was detected after 1 h at any IPTG concentration (Fig. 3a). At 3 h, band intensity correlated with IPTG concentration, with the strongest signal at 1 mM IPTG. After 6 h, maximum band intensity was again observed at 1 mM IPTG, followed by 0.4 mM, consistent with expression kinetics (Fig. 2b). At 26 °C, very low band intensity was observed after 1 h at all IPTG concentrations (Fig. 3b). By 3 h, moderate accumulation occurred at 0.4 and 1 mM IPTG, and by 6 h, comparable yields were observed across all IPTG concentrations, in agreement with kinetic fluorescence data (Fig. 2d).

### Cellular localisation of mCherry_p40_IBs

**Fig. 4:**
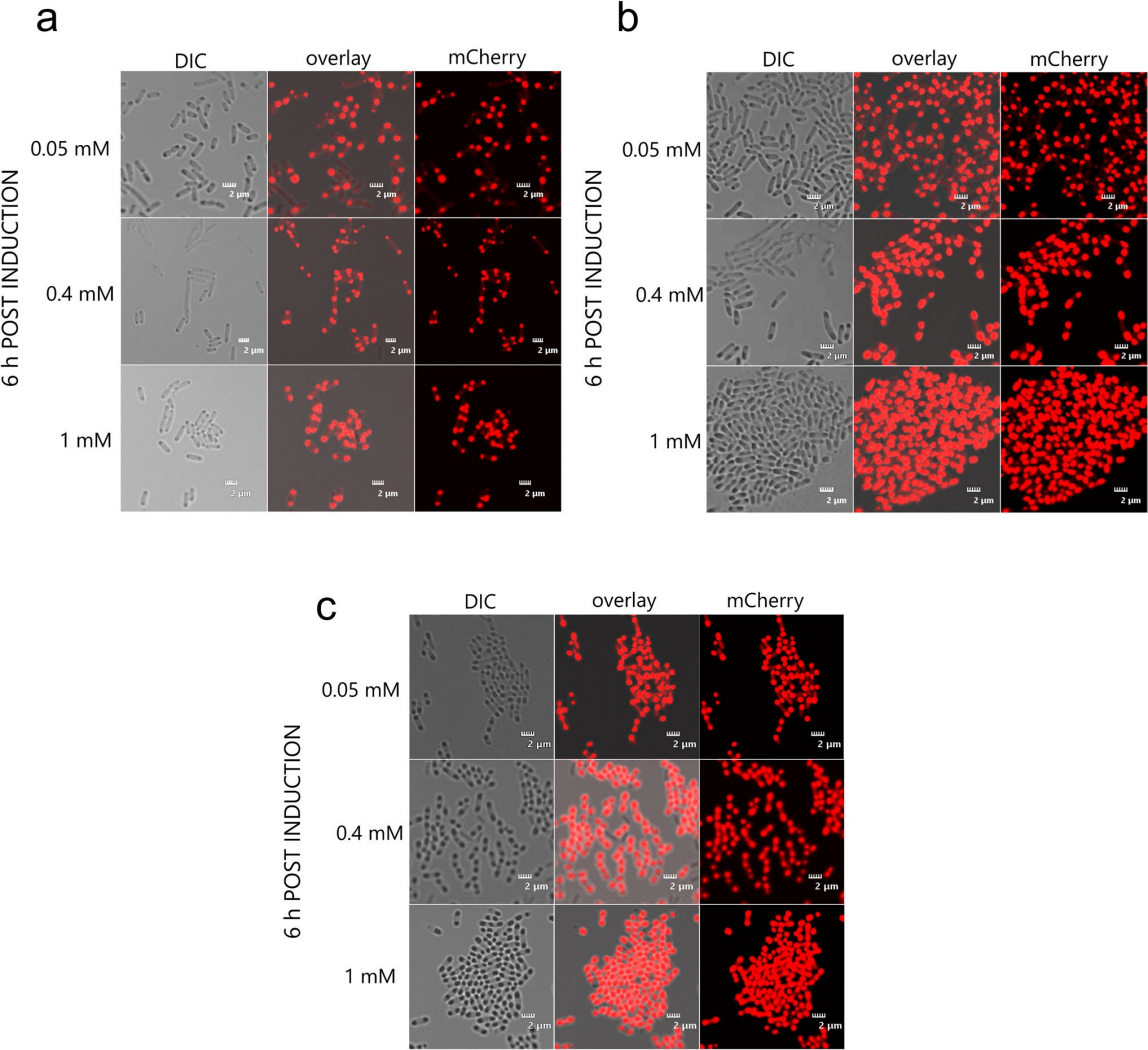
Confocal laser-scanning microscopy (CLSM) images of *E. coli* Tuner (DE3) cells expressing mCherry_p40_ IBs 6 h after induction at **a** 18 °C, **b** 26 °C, and **c** 37 °C

The intracellular distribution of mCherry_p40_ IBs was examined by CLSM under the same growth conditions and IPTG concentrations used for the expression-kinetics experiments. Samples were collected 6 h after induction and examined microscopically (Fig. 4).

At each growth temperature, CLSM images showed progressively larger fluorescent IB foci with increasing IPTG concentration. At 18 °C, cells induced with 0.05 mM IPTG exhibited smaller fluorescent foci than those observed at 0.4 and 1 mM IPTG (Fig. 4a). When cells were induced at 26 °C, the apparent size of the fluorescent foci also increased from 0.05 to 1 mM IPTG (Fig. 4b). At the optimal growth temperature of 37 °C, a similar trend was observed across the IPTG concentrations tested (Fig. 4c). CLSM revealed IPTG-dependent differences in the apparent size of fluorescent IB foci, even though the 6-h fluorescence-kinetics profiles (Fig. 2) and SDS–PAGE analyses of IB yield (Fig. 3) showed comparable final fluorescence intensity and protein levels across IPTG concentrations at 26 °C and 37 °C. In most cells, the fluorescent foci were located near the cell poles, consistent with previously reported localisation patterns of recombinant IBs in *E. coli*.

### Size and morphology of mCherry_p40_IBs

**Fig. 5:**
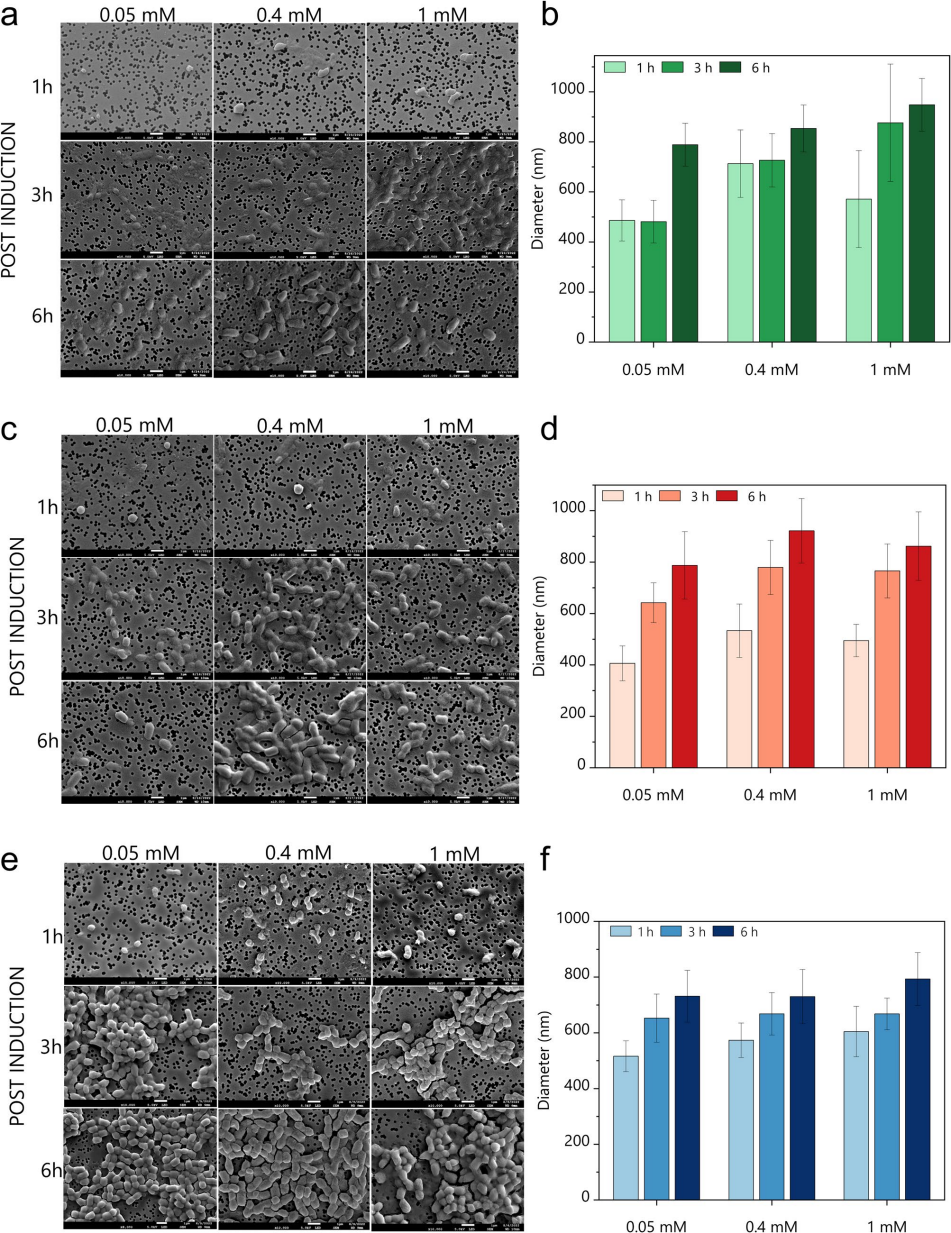
Field emission scanning electron microscopy (FESEM) images of purified mCherry_p40_ IBs obtained under various growth conditions at **a**18 °C. **c** 26 °C. **e** 37 °C (scale bar: 1 μm). Corresponding bar graphs show the calculated size distributions of IBs produced at **b**18 °C, **d** 26 °C, and **f** 37 °C, based on measurements of 50 individual IB particles per condition using ImageJ software. Aggregated clusters were excluded from the size analysis

The size and morphology of purified IBs obtained under all investigated growth conditions were analysed by FESEM. IB samples prepared as described in “Production of p40 IBs” section were deposited on 0.2-µm isopore membranes, gold-coated, and imaged to assess differences in IBs size and morphology. FESEM images showed clear variation in IB size and morphology as function of inducer (IPTG) concentration and induction time at each cultivation temperature. At 18 °C (Fig. 5a), IBs appeared cylindrical and slightly elongated, with lengths of 295–993 nm and widths of 272–660 nm. At 0.05 mM IPTG, IBs collected 6 h post-induction were elongated and fragile, whereas samples harvested after 1 h or 3 h at the same IPTG concentration contained IBs approximately half this size (Fig. 5b). IBs produced at 0.4 mM and 1 mM IPTG were larger than those formed at 0.05 mM IPTG at the corresponding induction times, with the largest structures (993 nm × 660 nm) observed after 6 h at 1 mM IPTG.

At 26 °C (Fig. 5c), IBs ranged from 550 to 977 nm in length and 517–663 nm in width and displayed both cylindrical and spherical morphologies. IB size increased progressively with induction time at all IPTG concentrations except for the 1 mM IPTG samples collected after 6 h, which were slightly smaller than those harvested after 3 h (Fig. 5d). Induction time also influenced IB shape: after 1 h at 0.05 or 0.4 mM IPTG, IBs were predominantly spherical, whereas after 3 h and 6 h, they appeared more cylindrical (Fig. 5c).

At the optimal growth temperature of 37 °C, IBs grew larger with increasing induction time at all IPTG concentrations (Fig. 5f). The particles tended to form clusters composed of slightly spherical structures after 1 h and more cylindrical aggregates after 3 h and 6 h (Fig. 5e). IBs ranged from 417 to 849 nm in length and 379–618 nm in width, with the largest observed after 6-h induction at 0.4 mM IPTG. Surfaces of IBs produced at 37 °C were visibly rougher than those obtained at lower temperatures. IB samples showing larger standard-deviation values reflected the non-homogeneous, polydisperse nature of the mCherryp_40_ IB population.

## Characterisation of different_p40_IBs constructs

### Enzyme_p40_

Three enzyme_p40_ IBs, namely Xyn_p40_, BactXyn_p40_, and BactAmy_p40_, were recombinantly expressed in *E. coli*, purified and characterised.

### Protein content

Protein content of each p40-based IBs was determined from 1 mg/mL (wet weight) samples (Fig. S6). mCherry_p40_ contained 72.8% target protein. Among the enzyme IBs, BactAmy_p40_ retained 80.47%, BactXyn_p40_ 71.6%, and Xyn_p40_ 64.3%. ZXR-2_p40_ IBs contained 49.0% target protein under similar cultivation conditions.

### Size and morphology

**Fig. 6:**
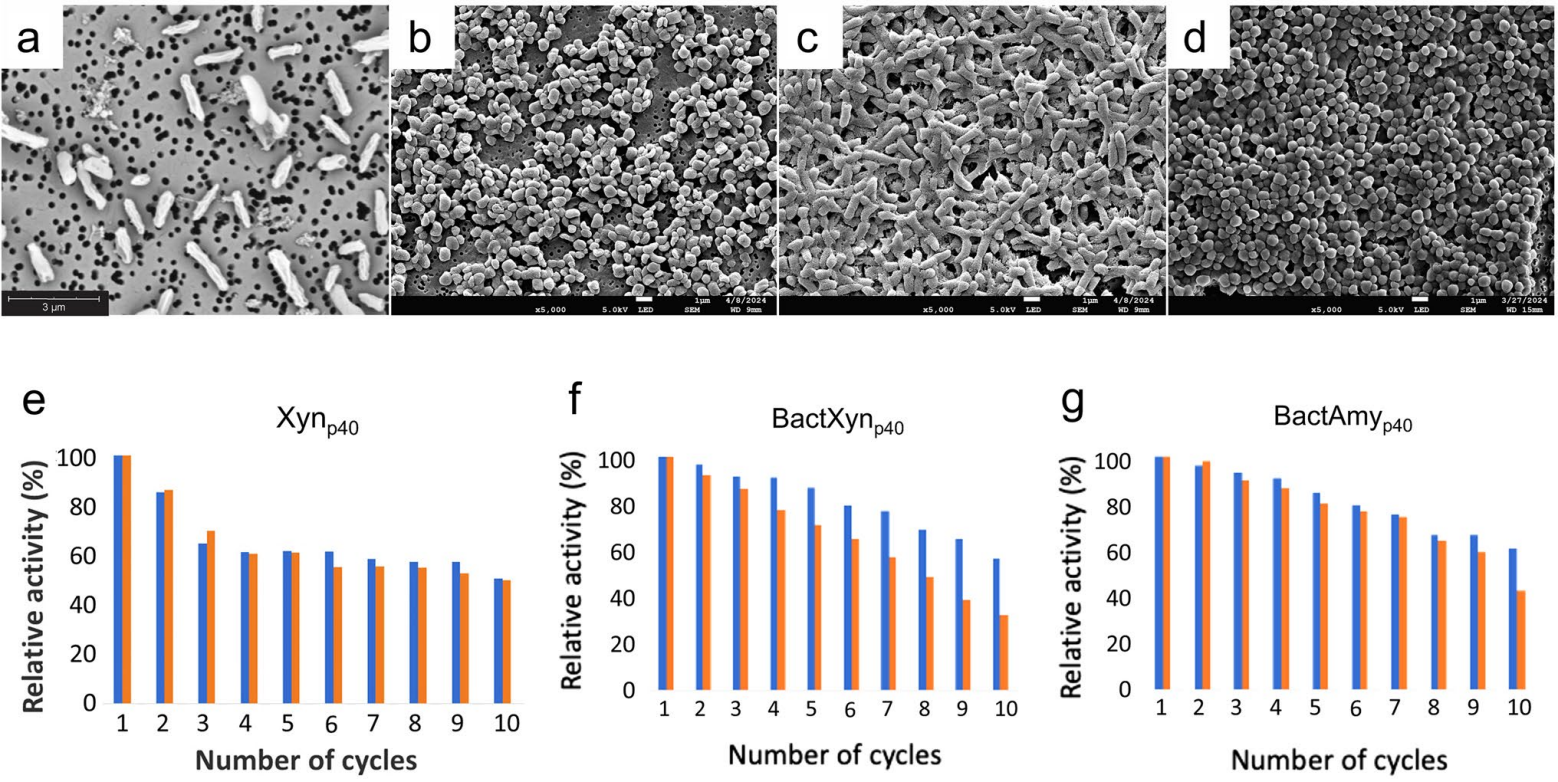
SEM images showing morphology differences among of various p40-based IBs. **a** Xyn_p40_. **b** BactXyn_p40_. **c** BactAmy_p40_. **d** Bare_p40_. Images were acquired at different magnifications to optimise visualisation of particle morphology; scale bars provide the spatial reference for comparison between samples. Enzyme recycling assays for insoluble enzyme-p40 IBs over ten consecutive reaction cycles (10 min each). **e** Xyn_p40_ at 70 °C (blue) and 80 °C (orange). **f** BactXyn_p40_ at 40 °C (blue) and 50 °C (orange). **g** BactAmy_p40_ at 70 °C (blue) and 80 °C (orange). Measurements were performed in duplicate. Standard deviation was less than ± 1

SEM showed that the enzyme fusion partner influenced IB morphology (Fig. 6). Bare_p40_ IBs formed smooth spherical particles (Fig. 6d). Xyn IBs were cylindrical and approximately 1.2 µm long (Fig. 6a). BactXyn IBs were smaller (~0.8 µm) and circular (Fig. 6b), while BactAmy_p40 _IBs were cylindrical and present in larger aggregates (Fig. 6c). BactAmy_p40_ IBs were cylindrical and present in larger aggregates (Fig. 6c). BactXyn_p40_ IBs were smaller (~ 0.8 µm) and circular (Fig. 6b), while Xyn_p40_ IBs were cylindrical and approximately 1.2 µm long (Fig. 6a).

### Enzyme_p40_IBs activity and recycling

The catalytic activities of Xyn_p40_, BactXyn_p40_, and BactAmy_p40_ IBs were measured using their respective substrates, oat spelt xylan for the xylanases, and potato soluble starch for the amylase. Assays were performed at each enzyme’s reported optimum temperature and at 10 °C below that optimum. All three enzyme_p40_ fusion proteins retained activity toward their specific substrates at both temperatures, and activity was linear with time and enzyme concentration.

Reusability was evaluated over ten consecutive 10-min reaction cycles (Fig. 6). Relative activity was expressed as a percentage of the initial activity (cycle 1). After ten cycles, each IB retained more than 50% of its initial activity. Xyn_p40_ showed no significant difference when assayed at 70 °C and 80 °C (Fig. 6e). BactXyn_p40_ (Fig. 6f) and BactAmy_p40_ (Fig. 6g) retained the highest relative activities, losing only 40–45% of their initial activity after ten cycles.

### Peptide_p40_

#### Antimicrobial activity of ZXR-2_p40_IBs

To assess the antimicrobial activity of ZXR-2_p40_ IBs, partially purified samples were tested at 1–12 µM against the dental-caries-associated bacteria *Lactobacillus fermentum* and *L. casei*. None of the concentrations tested achieved complete growth inhibition of either species (Fig. S7), although inhibition was more pronounced against *L. casei*. ZXR-2_p40_ was also tested against *Serratia marcescens* (Gram-negative) and *Staphylococcus epidermidis* (Gram-positive) at 2, 4, and 16 µM. The lowest concentration tested was sufficient to inhibit the growth of both organisms (data not shown), confirming that ZXR-2 peptide retained antimicrobial activity in the ZXR-2_p40_ IBs and was effective against selected Gram-positive and Gram-negative bacteria.

**Fig. 7:**
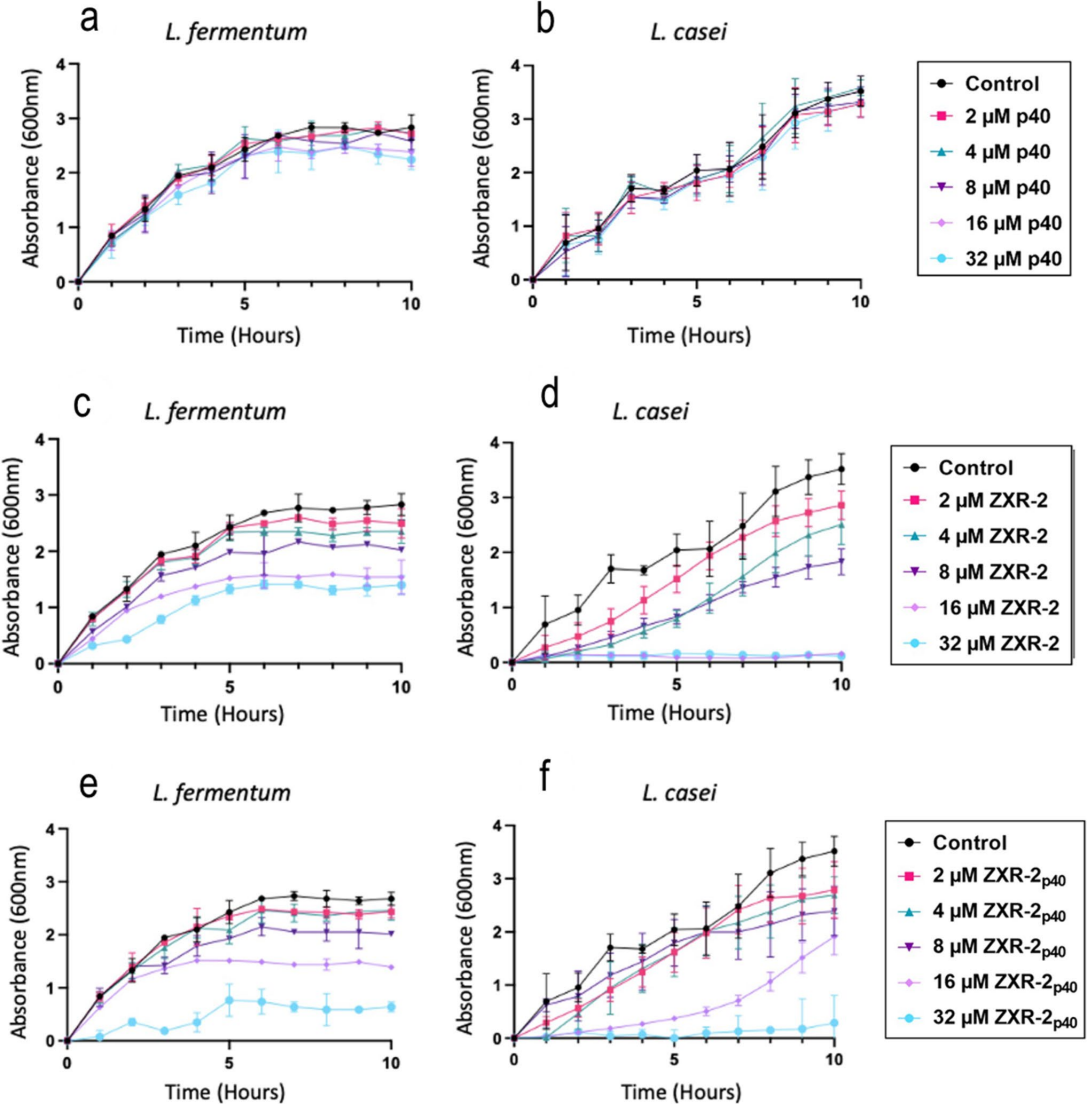
Effect of different concentrations (2–32 μM) of barep40, ZXR-2 peptide, and ZXR-2_p40_ on the growth of *L. fermentum *(**a**, **c**, **e**) and *L. casei* (**b**, **d**, **f**). Averages and standard deviation from at least three independent replicates are shown

A further comparison of ZXR-2_p40_, bare_p40_, and chemically synthesised ZXR-2 peptide was performed at 2–32 µM (Fig. 7). Bare_p40_ showed no effect on the growth of either *Lactobacillus* strains (Fig. 7a and b). The synthetic ZXR-2 peptide completely inhibited *L. casei* at 16 µM (Fig. 7d) and reduced *L. fermentum* OD_600_ by 54% after 10 h at 32 µM (Fig. 7c). ZXR-2_p40_ IBs was less effective against *L. fermentum,* with no complete inhibition at 32 µM (Fig. 7e). At this concentration, OD_600_ was reduced by 76.23% after 10 h (Fig. 7e). Complete inhibition of *L. casei* was observed at 32 µM of ZXR-2_p40_ (Fig. 7f). At 16 µM, ZXR-2_p40_ initially suppressed *L. casei* growth during the first 6 h but OD_600_ declined to only 45.7% reduction after 10 h.

**Fig. 8:**
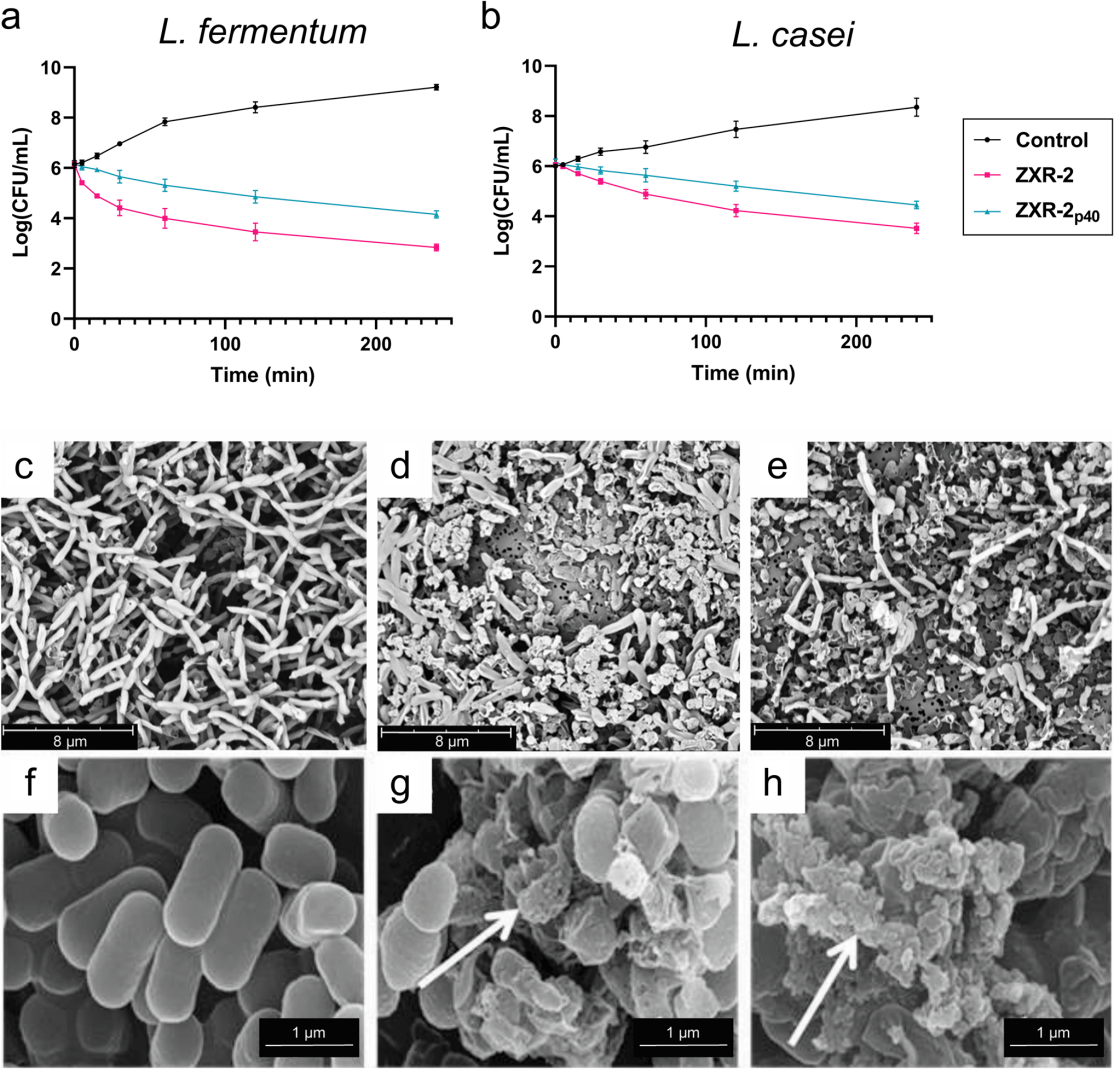
Viable count of *L. fermentum* (**a**) and *L. casei* (**b**) treated with 16 μM and 4 μM ZXR-2 and ZXR-2_p40_, respectively. Log CFU/ml values were plotted against time. Averages and standard deviation from at least three independent replicates are shown. SEM images of *L. casei* treated with 4 μM ZXR-2_p40_ under the same conditions used in the antimicrobial assay. Samples were collected at 0 h (**c**), 2 h (**d**), and 4 h (**e**). For comparison, images (**f**–**h**) show *L. fermentum* treated with the antimicrobial peptide ZXR-2 at 10 × MIC (20 μM), adapted from (Chen et al. 2017), with permission from Elsevier. These images provide a reference for peptide-induced membrane disruption. White arrows (**f**–**h**) indicate structural damage to the bacterial cells. Scale bars: **c**–**e**, 8 μm; **f**–**h**, 1 μm

Viable counts (CFU/mL) were used to quantify the bactericidal effect (Fig. 8). *L. fermentum* treated with 16 µM ZXR-2_p40_ or synthetic ZXR-2 peptide showed log reductions of 5.06 and 6.38, respectively, compared with untreated controls (Fig. 8a). For *L. casei*, treatment with 4 µM ZXR-2_p40_ and ZXR-2 peptide produced log reductions of 3.90 and 4.84, respectively (Fig. 8b). To correlate these antimicrobial effects with structural changes in bacterial cells, *L. casei* treated with 4 µM ZXR-2_p40_ under the same assay conditions was examined by SEM (Fig. 8c–e). Untreated cells (0 h) exhibited intact morphology with smooth cell surfaces (Fig. 8c). After 2-h exposure, early signs of surface disruption were observed (Fig. 8d). By 4 h, widespread cell shrinkage, membrane damage, and cell debris were evident (Fig. 8e), consistent with the bactericidal activity detected in the viable count assay.

### Cytotoxicity activity of ZXR-2_p40_

**Fig. 9:**
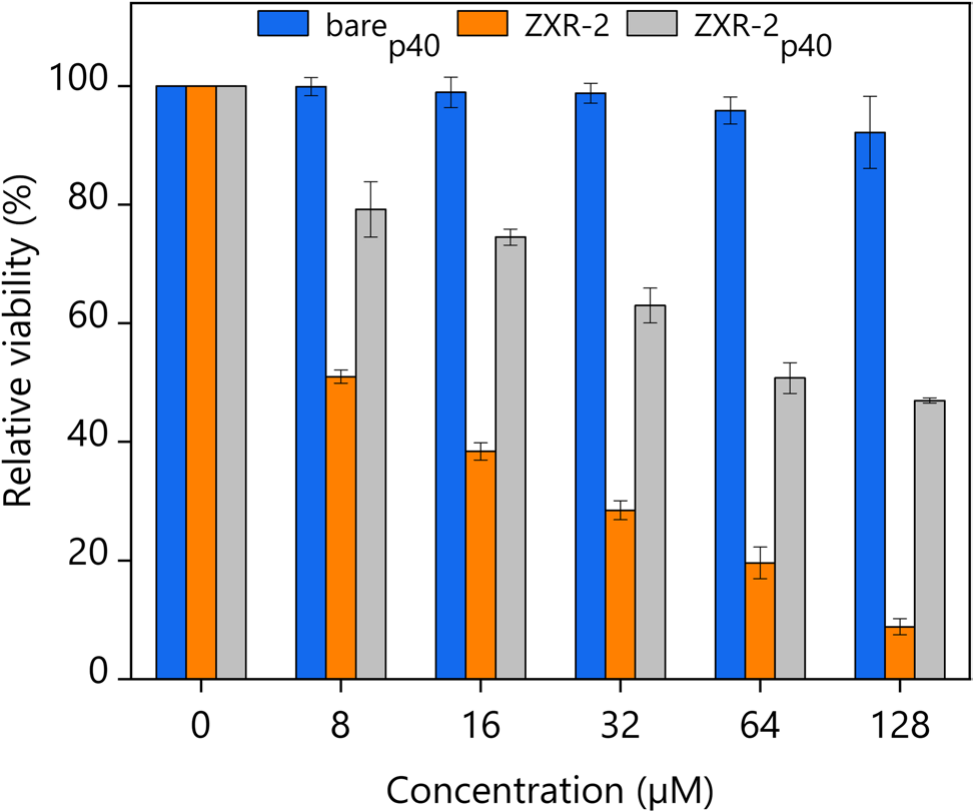
MTT assay of HEK293T cells treated with 0–128 μM of barep40 (blue), ZXR-2 peptide (orange), and ZXR-2_p40_ (grey). Averages and standard deviation from at least three independent replicates are shown

The cytotoxicity of ZXR-2_p40_ was assessed using the MTT assay with human embryonic kidney (HEK 293 T) cells. Cells were exposed to 0–128 µM of ZXR-2_p40_ and, for comparison, to equivalent concentrations of bare_p40_ and the synthetic ZXR-2 peptide (Fig. 9). Under these assay conditions, bare_p40_ produced no detectable cytotoxic effect. Both the synthetic ZXR-2 peptide and the ZXR-2_p40_ IBs reduced HEK293T cell viability in a concentration-dependent manner. At all concentrations tested, the synthetic ZXR-2 peptide showed at least 30% greater reduction in cell viability than ZXR-2_p40_.

## Discussion

IBs have traditionally been viewed as undesirable aggregates of misfolded proteins, yet mounting evidence has repositioned them as valuable functional biomaterials with applications in biocatalysis, protein delivery, and nanotechnology (García-Fruitós et al. 2012; Köszagová et al. 2022). In this context, the p40 domain was evaluated as a scaffold for producing IBs with distinct structural and functional properties. Computational predictions using I-TASSER modelling and the DichroWeb tool 2Struc (Miles et al. 2022), which generates a consensus across DSSP, STRIDE, and PSEA algorithms for protein secondary structure components, revealed a p40 architecture dominated by flexible loops, turns, and coils (58%), with substantial β-sheet content (24–32%) and minimal α-helices (16.2%), wherein the hydrophobic surfaces of these β-sheets, along with six Aggrescan-identified aggregation hotspots, drive intermolecular interactions underpinning its inherent self-assembly into ordered IBs (Fig. S1 and S2). The expression of bare_p40_ in *E. coli* BL21(DE3) yielded high levels of insoluble protein that could be efficiently purified using detergent-based extraction, with SDS–PAGE confirming the expected molecular weight. This straightforward production, which required no further optimisation, highlights the strong intrinsic aggregation propensity of p40 and its suitability for large-scale generation of recombinant IB.

DLS revealed that bare_p40_ IBs were highly polydisperse, with particle sizes ranging from 50 to 1000 nm in most suspension buffers, but as small as 10–40 nm when resuspended in Tris–HCl (pH 7.4). This condition also gave the lowest PDI (0.428), while the highest heterogeneity (PDI, 0.997) was observed in PBS (pH 6.0). These results indicate that the choice of resuspension buffer exerts a major influence on the aggregation state of isolated p40 IBs, enabling control of nanoparticle size distributions through physicochemical conditions rather than genetic modification. Morphological analysis with SEM imaging further confirmed the capacity of bare_p40_ to self-assemble into nanoscale and microscale structures. Particles observed in ultrapure water were predominantly spherical, ranging from 200 to 800 nm in diameter, consistent with the DLS data. Comparable observations were made in a study by (Gil-Garcia et al. 2020) who analysed ZapB-GFP IBs resuspended in water and reported an average size of 462.2 ± 69.51 nm by DLS with moderately polydisperse distributions. SEM revealed corresponding spherical assemblies. In a similar manner, bare_p40_ displayed broad size distributions dependent on suspension reagent, while SEM images visually confirmed its circular morphology in water. These findings support the interpretation of bare_p40_ IBs as self-assembling protein scaffold, highlighting the intrinsic capability of the p40 domain to drive nanoparticle formation.

The p40 domain demonstrated a strong capacity to drive IB formation across a wide range of induction conditions. mCherry_p40_ IBs formed robustly at IPTG concentrations from 0.05 to 1 mM and temperatures from 18 to 37 °C, even under conditions that usually favour soluble expression. Cultivation of *E. coli* at sub-optimal temperatures such as 18 °C or 26 °C is a widely used strategy to improve solubility and reduce aggregation (Schein and Noteborn 1988; Vera et al. 2007; Dragosits et al. 2011).

mCherry is a monomeric red fluorescent protein derived from *Discosoma* sp. and engineered from earlier DsRed variants to improve folding, maturation efficiency, and photostability (Shaner et al. 2004, 2005). It is widely used as a fluorescent reporter and fusion partner in recombinant protein studies. Recombinant expression of mCherry in *E. coli* under inducible systems has been reported in several studies, where functional fluorescence indicates successful intracellular folding of the reporter protein (Litcofsky et al. 2012). In addition, mCherry expression has been examined across different induction conditions, including IPTG induction and temperatures ranging from 18 to 37 °C, with analysis of soluble and insoluble protein fractions (Oscorbin et al. 2026). mCherry has also been used as a fusion partner to improve the stability and solubility of aggregation-prone enzymes in recombinant expression systems (Mestrom et al. 2019). Although aggregation can occur in specific fusion contexts, the available evidence indicates that this behaviour can depend strongly on the fused partner rather than reflecting an intrinsically aggregation-prone property of mCherry itself (Van der Henst et al. 2010). Accordingly, the robust IB formation observed for the mCherry_p40_ construct in this study is more plausibly associated with the aggregation-driving properties of the p40 scaffold.

In this study, mCherry_p40_ IBs still formed readily under these conditions, in contrast to other fusion systems. For example, GFP IBs have been reported to be strongly influenced by induction temperature and regime, with fluorescence often lost at elevated temperatures (Peternel et al. 2009). Bakholdina et al. (2021) reported similar temperature-dependent behaviour for GFP-based IBs, with fluorescence decreasing markedly at higher cultivation temperature. These results contrast with the current findings, where robust aggregation observed with mCherry_p40_ across a broad range of induction regimes highlighting the distinct behaviour of the p40 scaffold compared to other well-studied IB models. Comparison with alternative IB-inducing fusion systems further underscores the specific and robust contribution of the p40 domain. Jäger et al. (2019) employed mCherry as a model protein fused to the TDoT coiled-coil domain. Notably, insertion of a flexible linker ((GGGS)₃) plus Factor Xa cleavage site between TDoT and mCherry completely abolished IBs formation, with the fusion protein remaining almost entirely soluble (96.8% in the supernatant fraction). Only after deletion of the linker did the TDoT-mCherry construct produce IBs when expressed in auto-induction medium at 15 °C for 69 h. In striking contrast, the p40 fusion in the present study consistently and efficiently drove IB formation across a broad temperature range (18–37 °C) within a short 6-h cultivation period under standard expression conditions.

The yield of mCherry_p40_ IBs depended on post-induction temperature, IPTG concentration, and induction time. SDS-PAGE analysis showed that at 18 °C, accumulation of the 50.6 kDa fusion protein was slow, with no detectable protein band after 1 h, yet p40 still drove efficient IB formation after extended induction (6 h), underscoring its strong aggregation propensity. At 26 °C, a similar pattern was observed. At 37 °C, IB formation was rapid, with visible bands after 1 h at all IPTG levels despite slightly reduced cell density at higher IPTG. These results show that p40 efficiently drives IB formation even when induction is slow, while excessive IPTG at high temperature can slightly suppress biomass accumulation.

Similar relationships between induction temperature, IPTG concentration, and induction time have been reported previously for IB production in *E. coli* (Gutiérrez-González et al. 2019). Consistent with these observations, mCherryp40 IB formation was robust across the conditions tested here, although the balance between IPTG concentration and temperature influenced both the kinetics of fluorescence development and the final yield.

Cultivation parameters also influenced mCherry_p40_ purity. Lower cultivation temperatures appeared to reduced host-cell protein background and appeared to decrease contaminant proteins in IB preparations, consistent with reduced synthesis of outer-membrane proteins and phospholipids under sub-optimal growth (Rinas and Bailey 1992; Rinas et al. 1993). Similarly, Slouka et al. (2018) reported cleaner IBs produced after a 4-h induction at temperatures below 30 °C. This suggests that temperature not only determines IB yield but also impacts sample purity, an important consideration when IBs are to be developed as functional protein nanoparticles.

Microscopic analyses provided further insight into how cultivation parameters shape the architecture of mCherry_p40_ IBs. CLSM showed that mCherry_p40_ IBs consistently formed as discrete spherical bodies localised at the poles of *E. coli*, irrespective of temperature or inducer concentration. This polar localisation is a well-established feature of recombinant IBs, reflecting the association of aggregation with specific intracellular sites. For example (Wu et al. 2011), CLSM studies showed that GFP-ELK16 IBs appeared in *E. coli* as localised spherical bodies, while in soluble GFP expressing cells, the fluorescence was uniformly distributed in the cytoplasm. Similar CLSM studies by Raghunathan et al. (2014) found that the fluorescence of the insoluble GFP variant DL4 IBs was confined to one end of the cell whereas soluble GFP-hs1 displayed cytoplasmic fluorescence.

Interestingly, the CLSM data suggested that IB size tended to increase with IPTG concentration across all growth temperatures. This observation did not always align with the fluorescence kinetics and SDS–PAGE analyses, which indicated comparable final yields at different inducer concentrations. The discrepancy likely reflects that CLSM reports apparent aggregate size and distribution rather than total protein content, with larger but fewer aggregates possible at higher IPTG concentrations. Thus, these findings emphasise that IB morphology and size cannot always be inferred directly from bulk production data and highlight the need for complementary imaging approaches when evaluating IB properties. Because fluorophore maturation can be incomplete within aggregated states, particularly for recombinant proteins expressed as IBs in *E. coli*, CLSM fluorescence may under-estimate expressed mCherry relative to SDS–PAGE densitometry (García-Fruitós et al. 2005; Balleza et al. 2018).

High-resolution FESEM further confirmed the influence of cultivation parameters on IB size and morphology. The data indicated a general trend of increasing mCherry_p40_ IB size with longer induction times and higher IPTG concentrations, with morphological shifts from spherical to cylindrical forms at later stages. Similar induction-time dependent effects have been described for other proteins. Upadhyay et al. (2012) demonstrated that IB formation kinetics vary by protein: asparaginase IBs reached a narrow maximum size of 100–200 nm after a 4-h induction, while human growth hormone IBs ranged from 200 to 800 nm under the same conditions. This underlines that nucleation sites and aggregation tendencies are protein dependent. Slouka et al. (2018) likewise reported time-dependent IB growth, showing that toxic model proteins expressed with and without N-pro fusion formed IBs that enlarged from 400 nm at 4 h to 600 nm at 8 h, with further heterogeneity under prolonged cultivation. Achmüller et al. (2007) had previously demonstrated the utility of the N-pro fusion system for expressing diverse and difficult proteins, including the toxic gyrase inhibitor CcdB and the short 16-residue model peptide pep6His, as IBs in *E. coli*. In the present study, mCherry_p40_ IBs similarly increased in size with induction time, consistent with the principle that IB enlargement is strongly time dependent and varies with protein context.

In another study, Carrió et al. (2000) investigated the molecular basis of IB aggregation using β-galactosidase fused with aggregation-inducing VP1 tag from foot and mouth disease virus at either the N- or C-terminus (VP1LAC and LACVP1). Proteolytic digestion complemented by SEM showed that untreated IBs appeared as elongated, root-shaped structures with smooth surfaces, while digested IBs fragmented into sub-particles. Kinetic modelling revealed coexisting protein species with distinct protease sensitivities, confirming that IBs are heterogeneous assemblies of polypeptides in different conformational states. These findings support the concept that IBs are structurally complex and dynamic, aligning with the morphological heterogeneity observed in mCherry_p40_ IBs. Such variations across different culture conditions likely arise from culture-dependent effects on polypeptide accumulation and conformational states, which in turn modulate IB architecture and overall heterogeneity.

Fusion of p40 to three different enzymes, β-xylanases from *D. thermophilum* (Xyn_p40_) and *B. subtilis* (BactXyn_p40_), and the thermostable α-amylase from *B. licheniformis* (BactAmy_p40_), produced IBs whose size and morphology were dictated by the fusion partner. SEM showed that bare_p40_ IBs were smooth and spherical, whereas the three enzyme fusions exhibited distinct morphologies. BactAmy_p40_ IBs were cylindrical and tended to form larger aggregates, BactXyn_p40_ IBs were smaller and circular (0.8 µm), and Xyn_p40_ IBs were cylindrical in shape (1.2 µm). These differences likely reflect variations in molecular size and folding characteristics of the fusion partner, as has been observed previously with other enzyme IBs where aggregation domains dictate particle morphology (Georgiou and De Bernardez-Clark 1991; Morell et al. 2008; Peternel and Komel 2011).

Protein content analysis further underscored the effect of the fusion partner. Under identical cultivation conditions, BactAmy_p40_ IBs contained the highest proportion of target protein (80.5%), followed by BactXyn_p40_ (71.7%) and Xyn_p40_ (64.3%). These values are comparable to or higher than those reported for other functional enzyme IB systems, confirming efficient incorporation of the fusion partner into the aggregated fraction. By contrast, ZXR-2_p40_ IBs contained only 49.0% protein, suggesting that physicochemical properties such as size, hydrophobicity, or folding stability of the partner protein can significantly alter incorporation efficiency.

Importantly, all three enzyme_p40_ IBs retained catalytic activity. Xyn_p40_ and BactXyn_p40_ hydrolysed xylan, while BactAmy_p40_ degraded starch, with linear activity over time and enzyme concentration at both their reported optima and 10 °C below. Preservation of activity at sub-optimal conditions indicates that the IB format did not compromise the intrinsic thermostability of the enzymes (Morris et al. 1998; Rasiah and Rehm 2009; Yoon 2009; Abdel-Fattah et al. 2013). These findings support the view that enzyme IBs represent catalytically active IBs (CatIBs) (Kloss et al. 2018a, 2018b; Slouka et al. 2018; Jäger et al. 2019), rather than inactive aggregates.

Several of the catalytic enzymes used in this study have previously been reported as active recombinant proteins in *E. coli*, rather than as intrinsically aggregation-prone targets. For example, the GH11 xylanase from B*. subtilis* AMX-4 used here was previously cloned and expressed in *E. coli*, and the enzyme was purified from the cell-free extract of recombinant cells, indicating recovery of the protein from the soluble fraction (Yoon 2009). Similarly, the thermostable xylanase from *D. thermophilum* has been reported as a functional recombinant enzyme in heterologous expression systems (Morris et al. 1998). In addition, α-amylases from *B. licheniformis* have been described as soluble recombinant enzymes when expressed in *E. coli* (Gray et al. 1986). These reports indicate that the enzymes used in this study are generally handled as soluble recombinant proteins in the literature. Therefore, the consistent IB formation observed after fusion to p40 across these different enzymes further supports that aggregation is primarily driven by the p40 scaffold rather than by intrinsic aggregation tendencies of the catalytic domains. The p40-based α-amylase IBs displayed a high specific activity of 34.42 ± 0.39 U/mg (Vijayakumar and Sunna 2026), likely due to dense enzyme packing and enhanced stability facilitated by the p40 fusion. In contrast, α-amylase immobilised on polyester beads exhibited a lower specific activity of 0.506 U/mg (Rasiah and Rehm 2009), as surface immobilisation maintains enzyme kinetics but restricts overall performance through diffusion barriers or reduced enzyme density. Consequently, the p40-IB system provides ~ 68-fold greater activity, offering superior potential for scalable biocatalysis. Previous studies by Wu et al. (2011) similarly showed that IBs generated via fusion with the ionic self-assembling peptide ELK16 (LELELKLK)_2_ can retain very high levels of enzymatic activity. When ELK16 was genetically attached to the carboxyl terminus of amadoriase II (AMA) from *Aspergillus fumigatus* and β-xylosidase (XynB) from *Bacillus pumilus*, the insoluble IB fractions, AMA-ELK16 and XynB-ELK16, accounted for 87.5% and 94.4% of the total cellular activity, respectively. Notably, AMA-ELK16 IBs exhibited even higher specific activity (1875 U/mg) than the soluble AMA (1563 U/mg). These findings highlight that aggregation into IBs can stabilise or even enhance enzymatic performance, supporting the broader view that IBs are not inert deposits but structured, catalytically competent assemblies, consistent with the catalytic activity observed in enzyme_p40_ IBs.

Reusability assays confirmed that enzyme_p40_ IBs function as robust nanobiocatalysts. After ten reaction cycles at optimal temperature or 10 °C below, all IBs retained more than 50% of their initial activity. Xyn_p40_ showed no significant difference when assayed at 70 °C and 80 °C, while BactXyn_p40_ and BactAmy_p40_ were the most stable, losing only 40–45% after ten cycles.

These results compare favourably with other immobilisation strategies. For example, Care et al. (2017) reported that conventional XynB CLEAs rapidly lost stability, retaining less than 20% of their initial activity after a single cycle at 80 °C. In contrast, CLEAs of XynB fused to a silica-binding peptide (L-XynB CLEAs) retained around 50% activity after 12 cycles, and zeolite-bound L-XynB CLEAs maintained over 60% of their activity across the same period. Zeolite-bound L-XynB without CLEAs formation performed less well, dropping to ~ 30% activity after 12 cycles. Comparable results have also been described for other CatIBs. Krauss et al. (2017) reviewed numerous enzymes expressed as CatIBs that retained high activity and stability across multiple cycles without external carriers; Jäger et al. (2019) demonstrated that CatIB properties can be tailored to enhance recyclability; and Ölçücü et al. (2022) benchmarked CatIBs in flow chemistry, showing performance on par with immobilised enzymes. Overall, the enzyme_p40_ IBs described here exhibited stability and reusability comparable to classical immobilisation systems, with the added advantage of being produced directly in cells without external carriers. This positions them as recyclable carrier-free nanobiocatalysts that retain catalytic activity while allowing straightforward recovery of both the enzyme aggregates and the reaction products.

Due to the growing demand for antimicrobial peptides (AMPs) in clinical research, recombinant production is increasingly being explored as an alternative to chemical synthesis (Roca-Pinilla et al. 2020). However, AMP expression in bacterial hosts is often restricted by their inherent toxicity, which derives from their antimicrobial activity (Carratalá et al. 2020). To overcome limitations of toxicity, stability, and cost, AMPs are frequently fused to carrier proteins that stabilise the peptide and promote their accumulation as IBs (Deo et al. 2022). For example, JAMF1 nanoclusters, which incorporate a host defence AMP together with a bacterial-binding domain from gelsolin and the antimicrobial peptide sPLA₂, were successfully produced as IBs of ~ 500 nm and showed no detectable toxicity toward the production host (Roca-Pinilla et al. 2020). Similarly, the AMP GWH1 was fused at its N-terminus to scaffold proteins such as GFP and IFN-γ, yielding IB nanoparticles with bactericidal activity in vitro against *E. coli* (Carratalá et al. 2020).

Building on this strategy, we investigated whether the short antimicrobial peptide ZXR-2, previously shown to have both antimicrobial and cytotoxic properties (Zhou et al. 2016; Chen et al. 2017), could be recombinantly produced as functional IBs using p40 as a fusion partner. The resulting ZXR-2_p40_ IBs provide a model for assessing whether p40 can stabilise peptides with strong bioactivities and retain their functional properties in aggregated form.

ZXR-2_p40_ IBs retained antimicrobial activity, but their relative efficacy differed from the free synthetic peptide. In our assays, ZXR-2_p40_ inhibited *L. casei* more effectively than *L. fermentum*, whereas Chen et al. (2017) reported the opposite trend using clinical isolates. This discrepancy may stem from differences in strain origin. Chen et al. employed clinical isolates obtained from the Peking University School of Stomatology (Beijing, China), while our strains were sourced from IFM Quality Services (Sydney). As a result, the outcomes may not be directly comparable, reflecting natural variability in susceptibility among strains of the same species. Nevertheless, both the synthetic peptide and ZXR-2_p40_ showed measurable activity against *Lactobacillus*, confirming that the fusion format preserved the functional core of the peptide.

Importantly, ZXR-2_p40_ was also active against Gram-positive (*S. epidermidis*) and Gram-negative (*S. marcescens*) bacteria, demonstrating that the fusion did not abolish broad-spectrum activity. Direct comparisons, however, indicated that the free peptide was consistently more potent, producing greater growth inhibition and bactericidal effects in viable count assays. This likely reflects the higher solubility and membrane accessibility of the synthetic peptide compared with the aggregated IB form, in which a fraction of the peptide may be sterically constrained.

SEM analysis supported this interpretation, showing that ZXR-2_p40_ exposure led to visible damage of *L. casei* cells, including shrinkage, membrane disruption, and debris formation. These morphological alterations provide direct evidence that the IB-associated peptide retains the same mode of action as the free ZXR-2 peptide, namely disruption of bacterial membranes leading to cell death (Chen et al. 2017).

The cytotoxic potential of AMPs incorporated into IBs has previously been demonstrated. For example, Rothan et al. (2015) showed that anticancer peptides Tachiplicin I (TACH) and Latarcin 1 (LATA), when fused to the N- and C-terminus of the ribosome-inactivating protein MAP30, formed IBs that retained biological activity against cancer cells. In line with this, our study showed that ZXR-2_p40_ IBs preserved the cytotoxic effect of the peptide, albeit at reduced potency compared with the free synthetic form. Bare_p40_ itself produced no detectable cytotoxicity, confirming that the effect derived specifically from the ZXR-2 sequence. Both ZXR-2 and ZXR-2_p40_ reduced HEK293T cell viability in a dose-dependent manner, but across all concentrations, the IB form was consistently ~ 30% less effective. This attenuation is likely due to structural constraints or restricted peptide accessibility within IB aggregates, which limit direct interaction with cell membranes.

These findings highlight the ability of p40 to act as a stabilising carrier for peptides that are otherwise difficult to produce recombinantly due to toxicity, while still allowing retention of functional activity. However, the reduced potency relative to the free peptide suggests that accessibility is a key factor determining biological activity in peptide IBs. Unlike enzymes, where IBs themselves can function as reusable nanobiocatalysts, antimicrobial peptide IBs are unlikely to serve directly as pharmacological delivery forms. Instead, they may be more relevant as production and capture platforms, with active peptides released through engineered cleavage or mild solubilisation strategies. Although the present ZXR-2_p40_ preparations were produced in standard *E. coli* strains that carry lipopolysaccharide (LPS) and would require further engineering for therapeutic use, recent studies have shown that endotoxin-free ClearColi® strains can produce functional IBs and other bioactive proteins, including antigenic peptides for vaccine applications, without eliciting endotoxic responses (Rueda et al. 2014; Ueda et al. 2016; Chen et al. 2018). These developments open opportunities to explore p40-based fusions for safe large-scale production of bioactive peptides, including those with potential clinical applications.

## Conclusion

This study demonstrated the successful use of the p40 domain as a fusion partner for producing functional IBs from both enzymes and peptides. All tested enzyme_p40_ aggregates retained catalytic activity and exhibited notable stability and recyclability, underscoring their potential as robust carrier-free nanobiocatalysts. Fusion of the antimicrobial peptide ZXR-2 to p40 yielded IBs that preserved both antibacterial and cytotoxic activities, albeit at lower potency than the synthetic peptide, confirming that p40 can stabilise peptides that are otherwise challenging to express. Classical cultivation parameters such as temperature, induction time, and IPTG concentration allowed tunable production of p40 IBs, enabling control over size, morphology, and yield. These findings highlight the potential of biologically produced p40-based IBs as versatile nanomaterials for biotechnology. However, challenges remain, including host-related endotoxin contamination, process efficiency, and the economic scalability of IB production. Future research should address these issues while further exploring applications of p40-based IBs in therapeutic peptide development and industrial multi-enzyme biocatalysis.

## Supplementary Information

Below is the link to the electronic supplementary material.

ESM 1 (PDF 1.74 MB)

## Data Availability

All data supporting the findings of this study are available within the paper and its Supplementary Information
