# Supplementary material for: The p40 fusion domain as a scaffold for producing functional inclusion bodies
Source: Appl Microbiol Biotechnol. 2026 Apr 18;110:162. doi: 10.1007/s00253-026-13810-4 (PMC13219152; doi:10.1007/s00253-026-13810-4)
Supplement: Supplementary file 1 [file 253_2026_13810_MOESM1_ESM.pdf]

Supplementary Data

Submitted to Applied Microbiology and Biotechnology

The p40 Fusion Domain as a Scaffold for Producing Functional Inclusion Bodies

Santhosh Vijayakumar [1*], Riya Khetan [1*#], Anwar Sunna [1,2†]

1. School of Natural Sciences, Macquarie University, Sydney, NSW 2109, Australia.
2. Australian Research Council Industrial Transformation Training Centre for Facilitated Advancement of Australia's Bioactives (FAAB), Sydney, NSW 2109, Australia.

* These authors contributed equally to this work

# Current address:

Centre for Pharmaceutical Innovation (CPI), Clinical & Health Science, University of South Australia, Adelaide, SA 5000, Australia.

† Corresponding Author: Anwar Sunna

School of Natural Sciences

Macquarie University

North Ryde, NSW 2109

Australia

Email: anwar.sunna@mq.edu.au

Phone: +61 2 9850 4220

The secondary structure of the p40 from I-TASSER model **(Zhou et al. 2022)** was evaluated using 2Struc (https://2struccompare.cryst.bbk.ac.uk/index.php) **(Miles et al. 2022)** to generate a consensus across four algorithms: DSSP (H-bond based), STRIDE (geometry/angle based), PSEA (sequence based), The structural composition was consistent across all assessment methods, revealing an architecture dominated by loops, β-turns and coils (58%) (Fig. S1). This prevalence of flexible, unordered regions is characteristic of LPMOs **(Tamburrini et al. 2021)**. Among ordered structures, β-sheets constitute the primary component, ranging from 24.1% (DSSP) to 32% (PSEA); notably, algorithms incorporating torsional angles (STRIDE and PSEA) identified higher β-content by detecting extended strands excluded by the stricter hydrogen-bond criteria of DSSP. In contrast, α-helices represent a minor structural component (16.2%). The substantial β-sheet content (24-32%) is critical to p40's behavior of inherent self-aggregation. AA10 LPMOs typically possess a rigid β-sandwich core **(Forsberg et al. 2019; Forsberg et al. 2025)**. In p40, these sheets likely expose hydrophobic surfaces that drive intermolecular β-interactions, leading to the formation of ordered IBs. Moreover, AGGRESCAN analysis identified seven aggregation hotspots within p40 (Fig. S2), likely mapping to β-sheet regions enriched with high-propensity residues **(Conchillo-Solé et al. 2007)**. These residues likely transform the β-strands into nucleation seeds for IB assembly, providing a physical explanation for the carrier-free immobilisation potential of the p40 system.

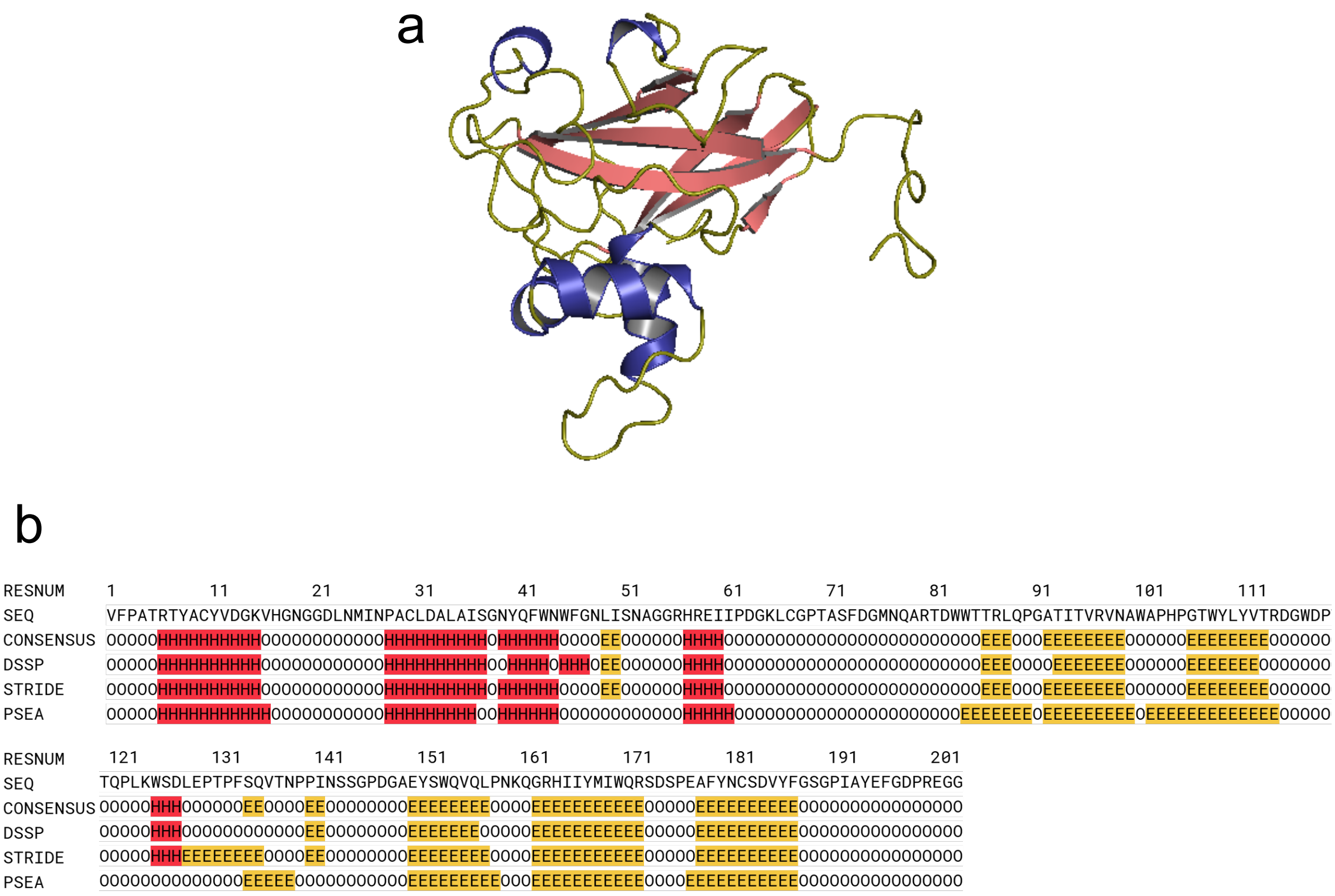

Fig. S1 (a) I-TASSER modelled three-dimensional structure of p40 domain. (b) Summary of p40 domain secondary structure predictions using 2StrucCompare tool from DichroWeb. The amino acid sequence is shown at the top, with multiple rows below indicating predicted structures: H (red) for α-helix, E (yellow) for β-sheet, and O for other components

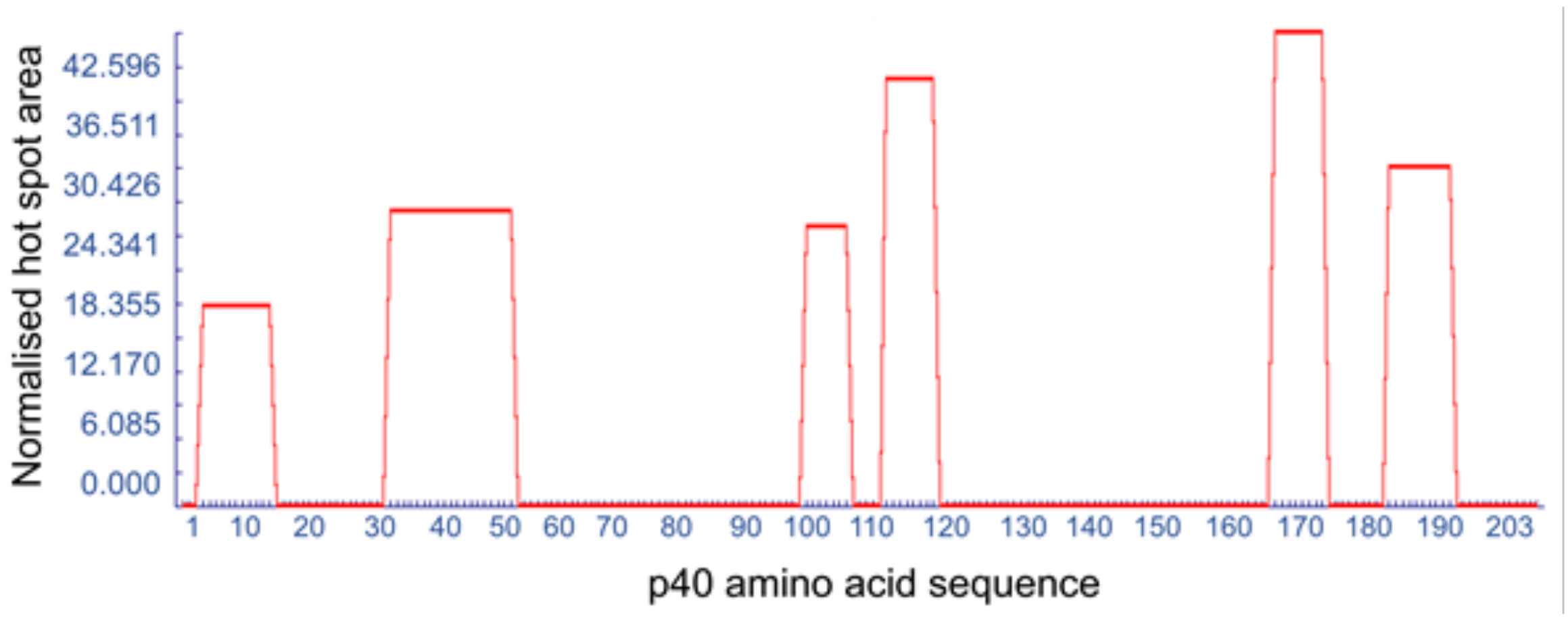

Fig. S2 Representative plots illustrating aggregation-prone hotspots in the p40 protein sequence predicted by AGGRESCAN computational tools

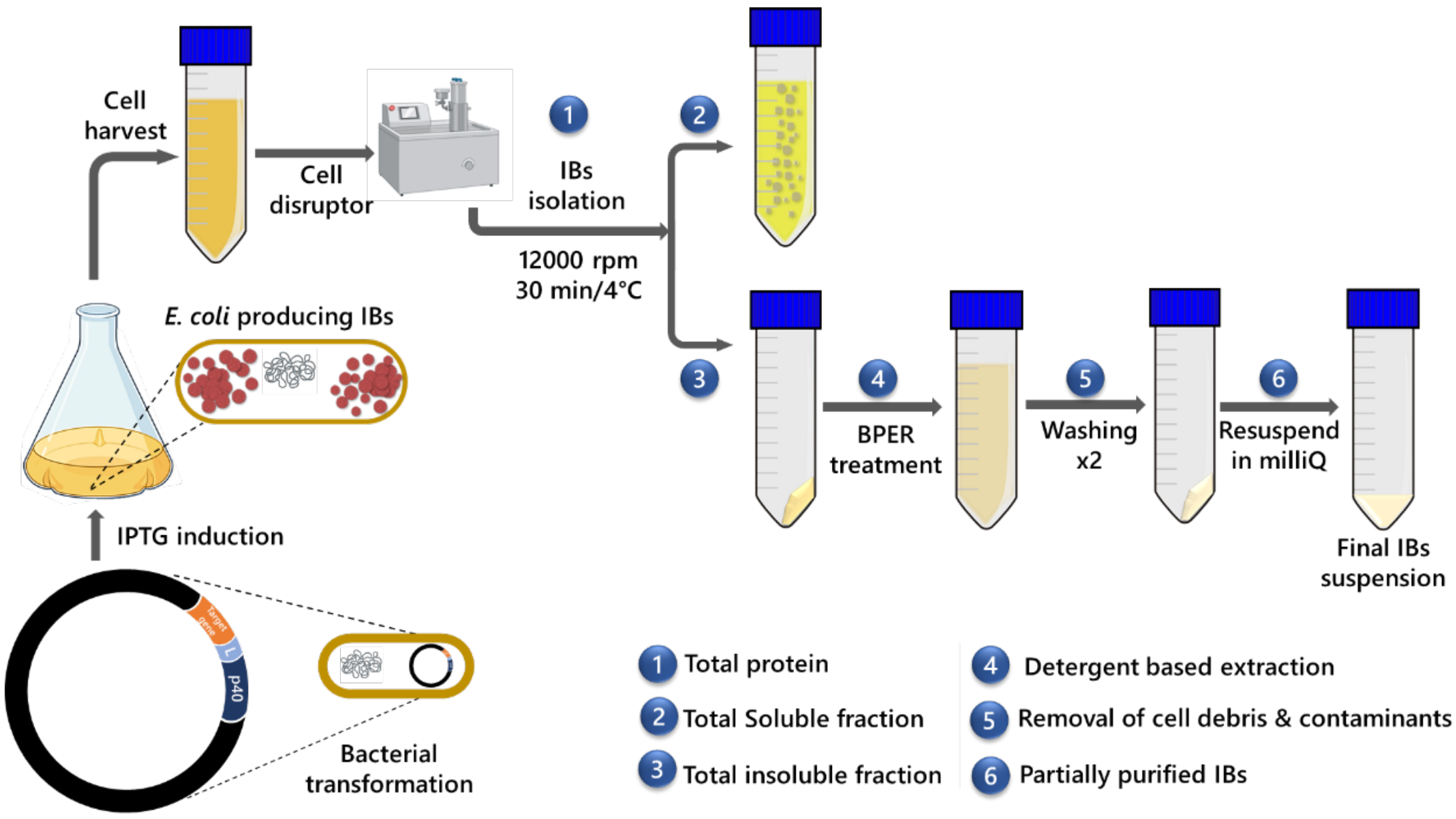

Fig. S3 Diagrammatic representation of standard method for the expression and purification of recombinant IBs. Numbers indicate the sample collected at each stage of purification.

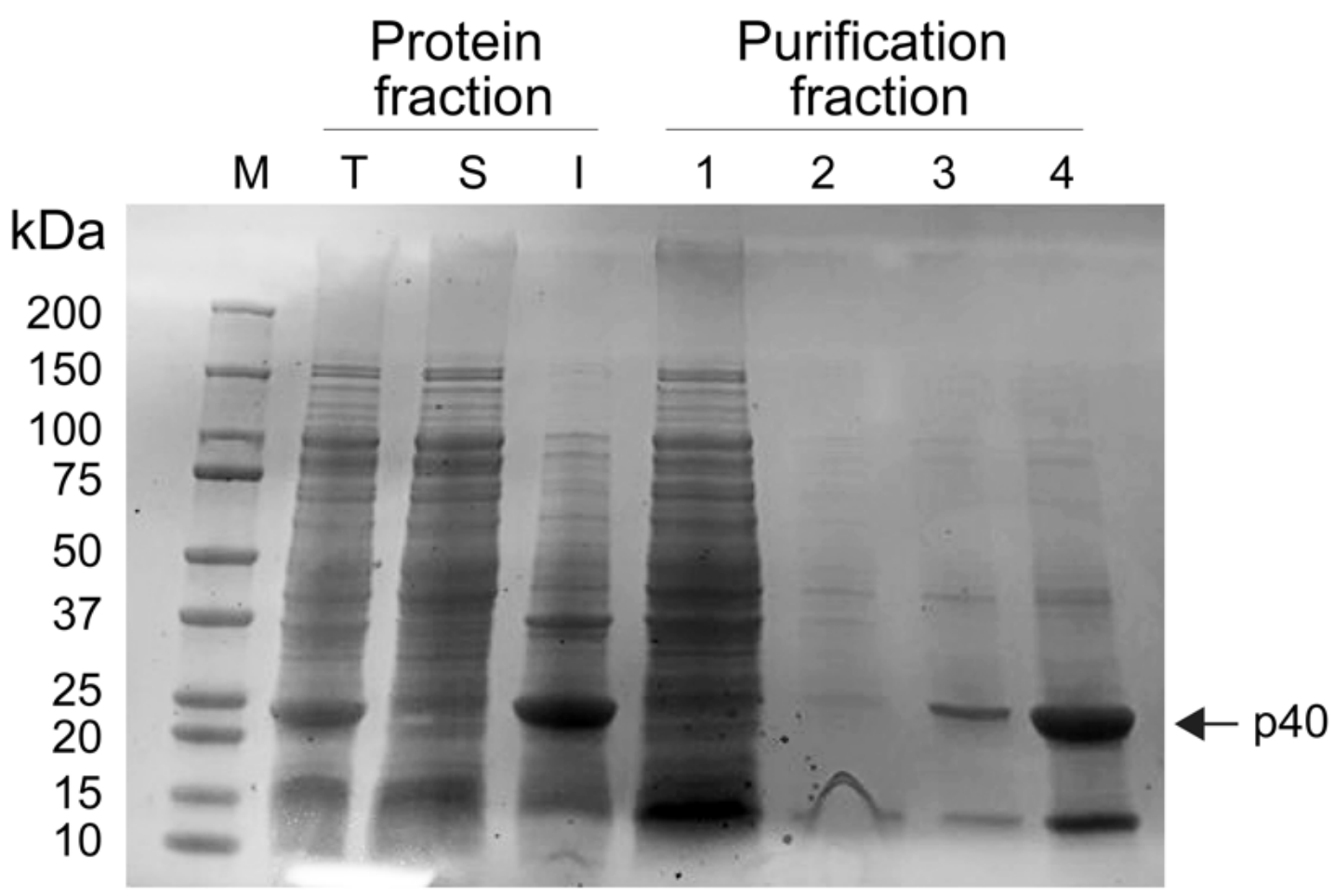

Fig. S4 Coomassie blue stained SDS–PAGE analysis of bare$_{p40}$ IBs after expression in *E. coli* and partial purification. Lane assignments are as follows: M, molecular weight marker; T, total protein fraction; S, soluble fraction; I, insoluble fraction; 1, supernatant after first diluted B-PER wash; 2, supernatant after second diluted B-PER wash; 3, final washed bare$_{p40}$ pellet (diluted 1:10); 4, final washed bare$_{p40}$ pellet (undiluted). Partially purified bare$_{p40}$ protein band was observed at ~24.47kDa. Precision plus dual colour protein standard was used as molecular weight marker.

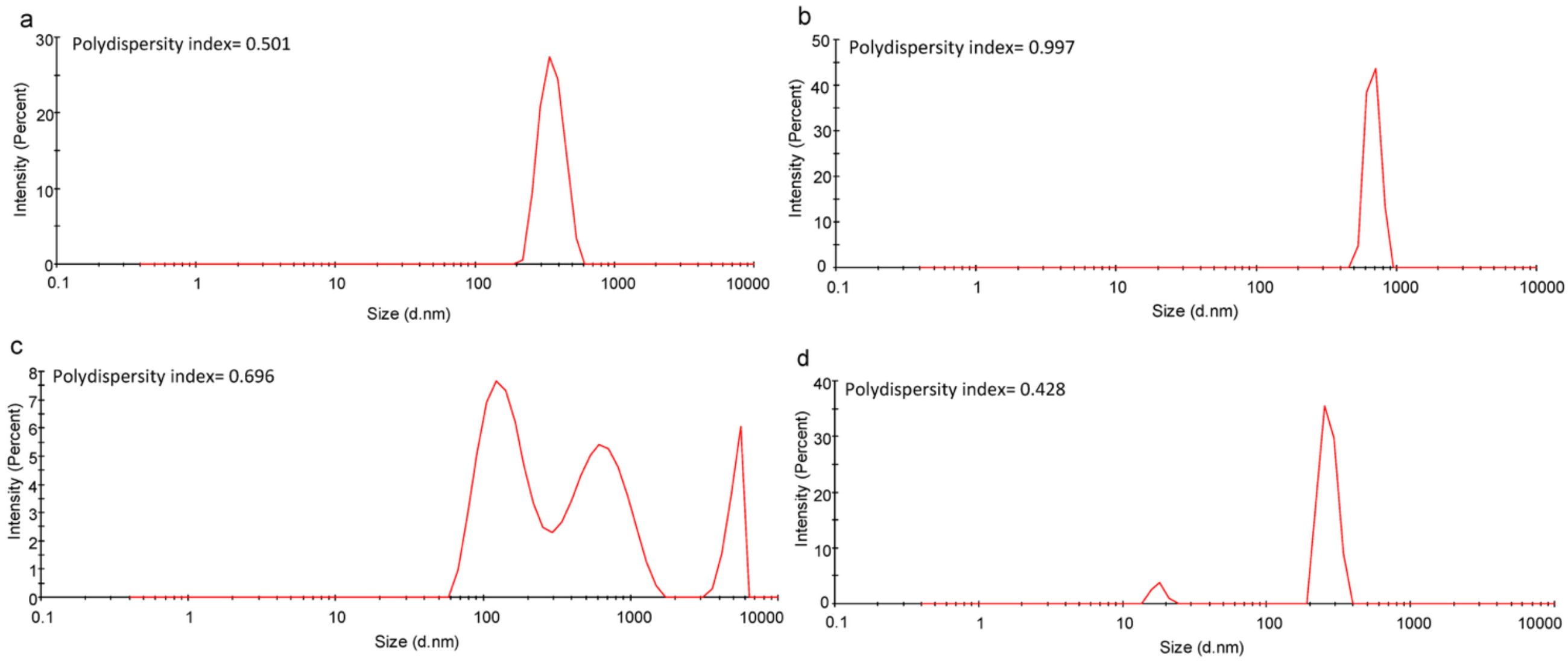

Fig. S5 Intensity plot of Dynamic light scattering (DLS) of barep40 resuspended in four different solutions. (a) Ultrapure water; (b) 50mM PBS, pH 6.0; (c) 5mM HCl, pH 4.1 and (d) 10mM Tris-HCl, pH 7.4.

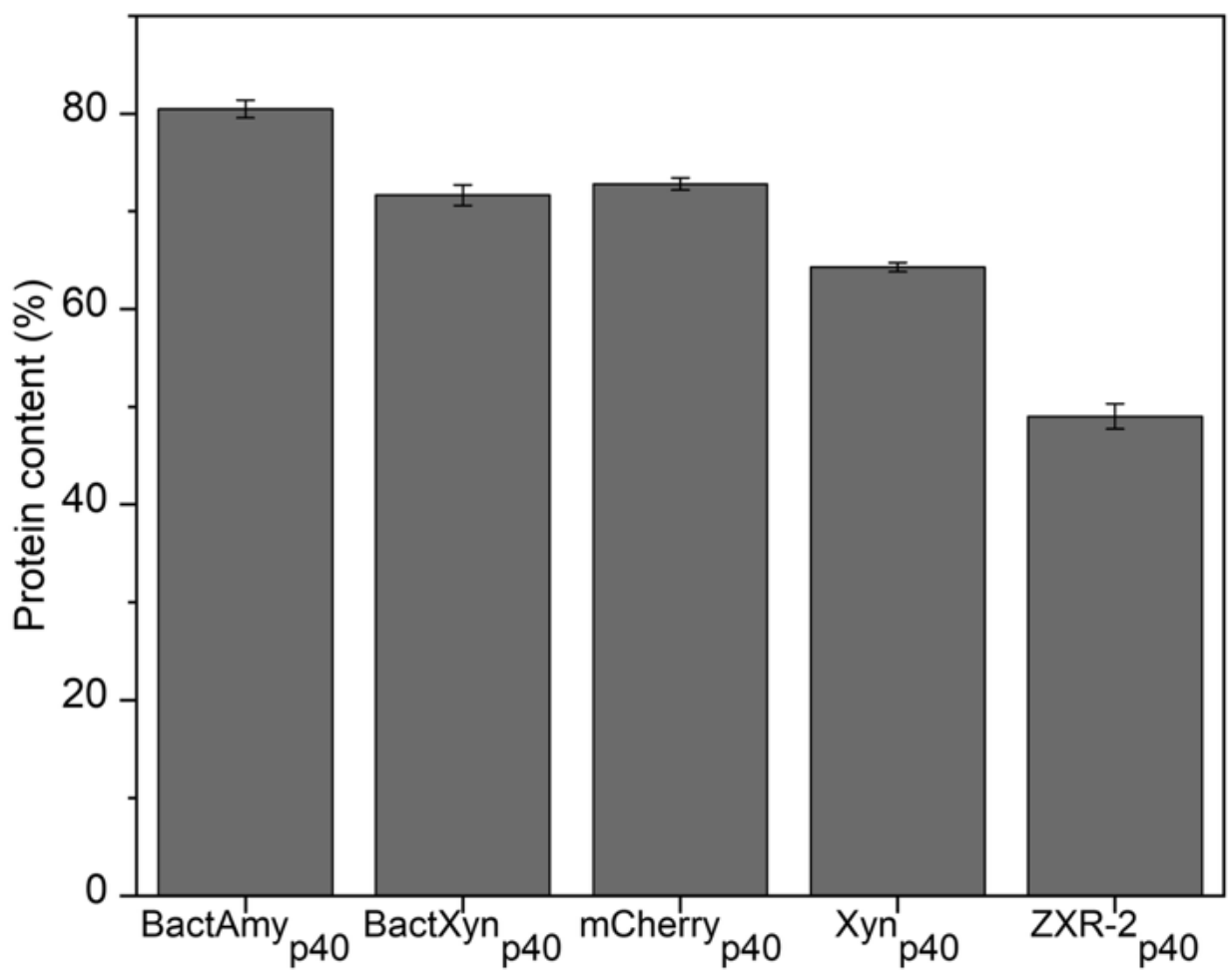

Fig. S6 Protein content estimation of various partially purified p40-based IBs.

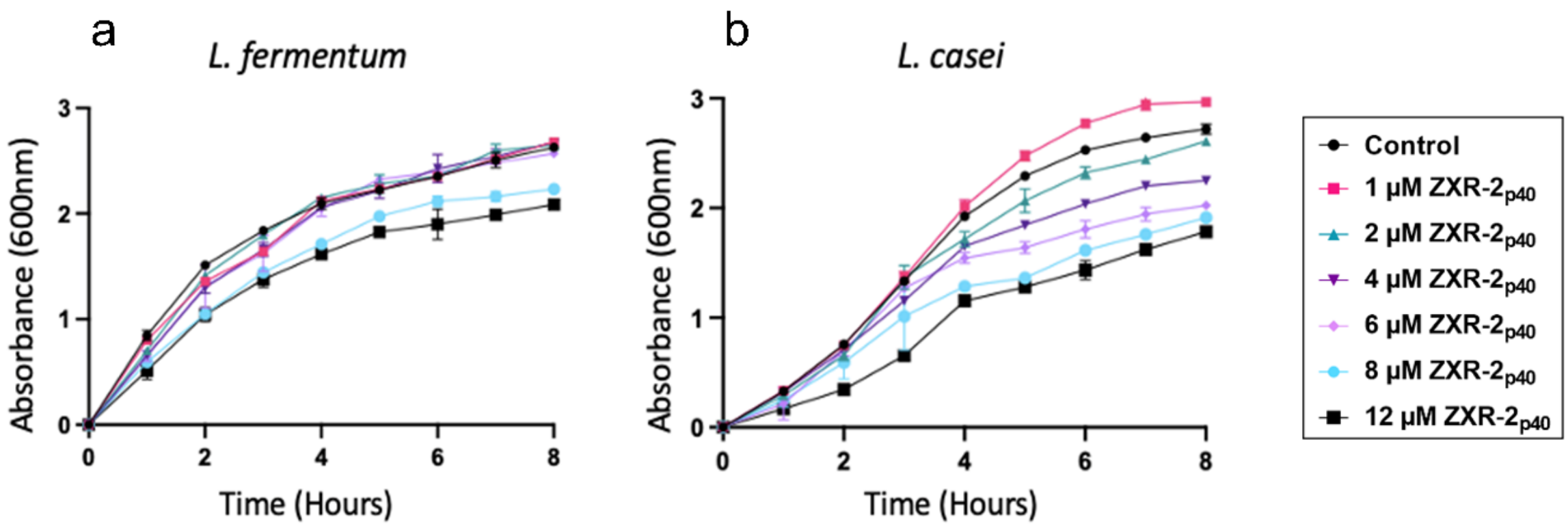

Fig. S7 Effect of different concentrations (1-12 µM) of ZXR-2_{p40} on the growth of *L. fermentum* (a) and *L. casei* (b). Averages and standard deviation from at least three independent replicates are shown

Fig. S8 Raw gel image – Figure 3

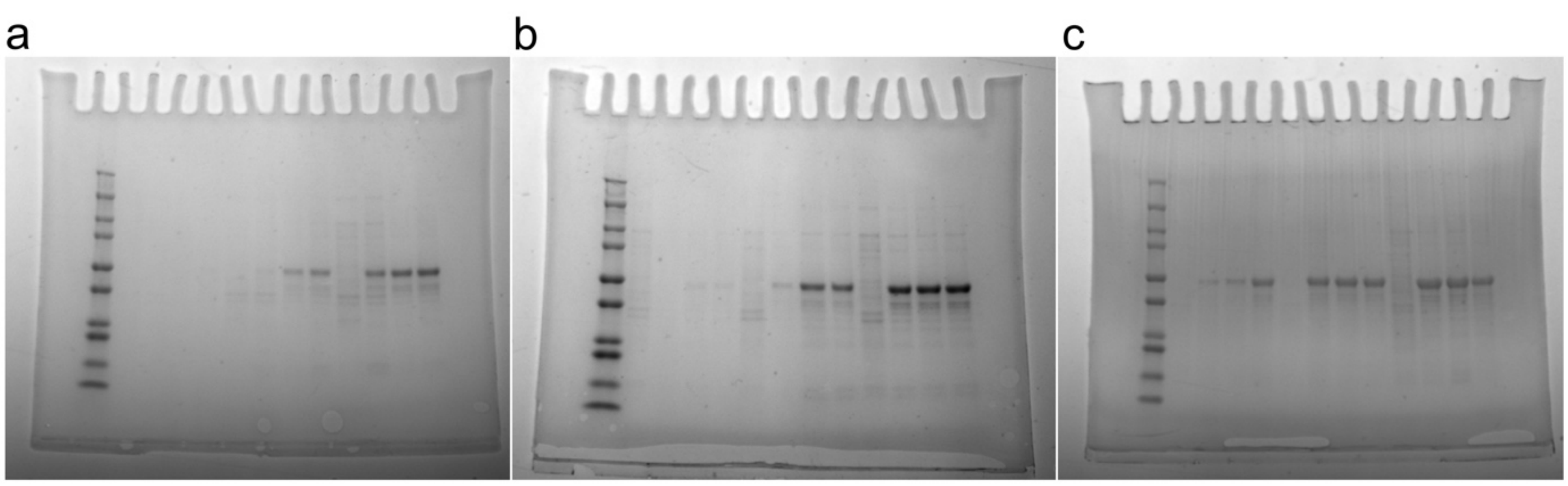

Table S1. Inserts used for the construction of p40 fusion proteins. The table summarises the source organism of each insert, flanking restriction sites used for cloning into the p40 fusion construct, protein family classification, functional annotation, and reported biological or catalytic activity.

Inserts	Source organism	Flanking sites	Construction	Family	Function	Bio/catalytic activity
mCherry	*Discosoma sp*	NcoI-HindIII	mCherry L p40	Fluorescent protein-related	Red fluorescence	yes
BactAmy	*Bacillus licheniformis*	NcoI-HindIII	bactAmy L p40	GH family 13	Hydrolyse starch	yes
BactXylB	*Bacillus subtilis*	NcoI-HindIII	bactXylB L p40	GH family 11	Hydrolyse xylan	yes
XynB	*Dictyoglomus thermophilum*	NcoI-HindIII	XynB L p40	GH family 11	Hydrolyse xylan	yes
ZXR-2	*Androctonus mauritanicus*	PciI-EcoRI	ZXR-2 L p40	Lytic peptide	antibacterial	yes

References

Conchillo-Solé O, De Groot NS, Avilés FX, Vendrell J, Daura X, Ventura S (2007) AGGRESCAN: a server for the prediction and evaluation of “hot spots” of aggregation in polypeptides. BMC Bioinformatics 8:65. https://doi.org/10.1186/1471-2105-8-65

Forsberg Z, Sørlie M, Petrović D, Courtade G, Aachmann FL, Vaaje-Kolstad G, Bissaro B, Røhr ÅK, Eijsink VG (2019) Polysaccharide degradation by lytic polysaccharide monooxygenases. Curr Opin Struct Biol 59:54–64. https://doi.org/10.1016/j.sbi.2019.02.015

Forsberg Z, Tuveng TR, Eijsink VGH (2025) A modular enzyme with combined hemicellulose-removing and LPMO activity increases cellulose accessibility in softwood. FEBS J 292:75–93. https://doi.org/10.1111/febs.17250

Miles AJ, Ramalli SG, Wallace BA (2022) DICHROWEB, a website for calculating protein secondary structure from circular dichroism spectroscopic data. Protein Sci 31:37–46. https://doi.org/10.1002/pro.4153

Tamburrini KC, Terrapon N, Lombard V, Bissaro B, Longhi S, Berrin J-G (2021) Bioinformatic analysis of lytic polysaccharide monooxygenases reveals the pan-families occurrence of intrinsically disordered c-terminal extensions. Biomolecules 11:1632. https://doi.org/10.3390/biom11111632

Zhou X, Zheng W, Li Y, Pearce R, Zhang C, Bell EW, Zhang G, Zhang Y (2022) I-TASSER-MTD: a deep-learning-based platform for multi-domain protein structure and function prediction. Nat Protoc 17:2326–2353. https://doi.org/10.1038/s41596-022-00728-0
